# Supplementary material for: Association between self-reported alcohol consumption and diastolic dysfunction: a cross-sectional study
Source: BMJ Open. 2023 Oct 11;13(10):e069937. doi: 10.1136/bmjopen-2022-069937 (PMC10583109; doi:10.1136/bmjopen-2022-069937)
Supplement: Author's manuscript [file bmjopen-2022-069937.draft_revisions.pdf]

BMJ Open is committed to open peer review. As part of this commitment we make the peer review history of every article we publish publicly available.

When an article is published we post the peer reviewers' comments and the authors' responses online. We also post the versions of the paper that were used during peer review. These are the versions that the peer review comments apply to.

The versions of the paper that follow are the versions that were submitted during the peer review process. They are not the versions of record or the final published versions. They should not be cited or distributed as the published version of this manuscript.

BMJ Open is an open access journal and the full, final, typeset and author-corrected version of record of the manuscript is available on our site with no access controls, subscription charges or pay-per-view fees (http://bmjopen.bmj.com).

If you have any questions on BMJ Open's open peer review process please email info.bmjopen@bmj.com

BMJ Open

Association between self-reported alcohol consumption and diastolic dysfunction, a cross-sectional study

Journal:	*BMJ Open*
Manuscript ID	bmjopen-2022-069937
Article Type:	Original research
Date Submitted by the Author:	07-Nov-2022

Complete List of Authors:	Daka, Bledar; University of Gothenburg Sahlgrenska Academy, Medicine<br>Bennet, Louise; Lund University, Family Medicine, Deaprtment of Clinical Sciences<br>Råstam, Lennart ; Lund University, Department of Clinical Sciences, Malmö, Sweden<br>Hellgren, Margareta; University of Gothenburg<br>Li, Ying; Gothenburg University Library<br>Magnusson, Martin; Lund University<br>Lindblad, Ulf; Lund University, Department of Clinical Sciences in Malmö

Keywords:	Echocardiography < CARDIOLOGY, PREVENTIVE MEDICINE, Heart failure < CARDIOLOGY

SCHOLARONE™
Manuscripts

I, the Submitting Author has the right to grant and does grant on behalf of all authors of the Work (as defined in the below author licence), an exclusive licence and/or a non-exclusive licence for contributions from authors who are: i) UK Crown employees; ii) where BMJ has agreed a CC-BY licence shall apply, and/or iii) in accordance with the terms applicable for US Federal Government officers or employees acting as part of their official duties; on a worldwide, perpetual, irrevocable, royalty-free basis to BMJ Publishing Group Ltd ("BMJ") its licensees and where the relevant Journal is co-owned by BMJ to the co-owners of the Journal, to publish the Work in this journal and any other BMJ products and to exploit all rights, as set out in our licence.

The Submitting Author accepts and understands that any supply made under these terms is made by BMJ to the Submitting Author unless you are acting as an employee on behalf of your employer or a postgraduate student of an affiliated institution which is paying any applicable article publishing charge ("APC") for Open Access articles. Where the Submitting Author wishes to make the Work available on an Open Access basis (and intends to pay the relevant APC), the terms of reuse of such Open Access shall be governed by a Creative Commons licence – details of these licences and which Creative Commons licence will apply to this Work are set out in our licence referred to above.

Other than as permitted in any relevant BMJ Author's Self Archiving Policies, I confirm this Work has not been accepted for publication elsewhere, is not being considered for publication elsewhere and does not duplicate material already published. I confirm all authors consent to publication of this Work and authorise the granting of this licence.

For peer review only

Association between self-reported alcohol consumption and diastolic dysfunction, a cross-sectional study

Bledar Daka MD MD PhD[1], Louise Bennet MD, PhD[2, 3], Lennart Råstam MD, PhD[2], Ying Li PhD[1], Margareta I Hellgren MD PhD [1, 5], Martin Magnusson MD PhD[2,6, 7, 8], Ulf Lindblad MD, PhD[1, 5]

[1] Family medicine, School of Public Health and Community Medicine, The Sahlgrenska Academy, the University of Gothenburg, Sweden
[2] Lund University, Department of Clinical Sciences, Malmö, Sweden
[3] Clinical Trial Unit, Skåne University Hospital, Lund, Sweden
[4] Department of Cardiology, Skaraborg Hospital, Skövde, Sweden
[5] Skaraborg Institute, Skövde, Sweden
[6] Department of Cardiology, Skåne University Hospital, Malmö, Sweden
[7] Hypertension in Africa Research Team (HART); North-West University, Potchefstroom, South Africa
[8] Wallenberg Center for Molecular Medicine, Lund University, Lund, Sweden

Correspondence to: Bledar Daka Associated Professor, School of Public Health and Community Medicine/Primary Health Care, Hälsovetarbacken 7, Box 454, SE 405 30 Göteborg, Sweden
Phone: +46 31 786 68 31
e-mail: bledar.daka@gu.se

For peer review only

Abstract

Background and objectives: While alcohol consumption is associated with common risk factors for diastolic dysfunction the independent impact of low to medium-high levels of alcohol consumption on this condition in a community setting is still unclear.

Thus, the aim of this study was to explore this association in a representative population sample employing optimal echocardiographic techniques.

Design: Cross-sectional observational study in community-based population

Settings, participants, and methods: Men (500) and women (538) 30 to 75 years (Mean age 55.4±13) were consecutively invited to a physical exam, interview, conventional echocardiography, including Tissue Velocity Imaging. Diastolic dysfunction was defined according to the European Society of Cardiology criteria, excluding subjects with ejection fraction <45%, self-reported history of heart failure, or atrial fibrillation on ECG. Self-reported alcohol intake using a validated questionnaire was categorized as *no intake, low and medium-high* intake.

Results: In total, 500 men and 538 women were successfully examined. Diastolic dysfunction was identified in 16% (79/500) of the men and 13% (58/538) of the women, p=0.037. The multivariable adjusted model revealed a strong and independent association between alcohol intake and diastolic dysfunction. In fact, using no alcohol intake as reference, diastolic dysfunction was independently associated with alcohol consumption in a dose-dependent fashion; *low consumption,* OR 2.3 (95% CI 1.3 to 4.0) and *medium-high consumption OR* 3.1 (95% CI 1.6 to 6.2), respectively.

Conclusion: There was a significant association between alcohol consumption and diastolic dysfunction starting already low levels that was supported by a dose-dependent pattern. These results are important in public health policies to reduce alcohol consumption.

Keywords: *diastolic dysfunction, echocardiography, heart failure, alcohol consumption*, public health

For peer review only - http://bmjopen.bmj.com/site/about/guidelines.xhtml

Strengths and limitations of the study

1. Echocardiography was performed in a large population-based cohort using standardized methods. The re-evaluation of a sub-sample by a senior cardiologist confirmed the first assessment and the consistency.
2. Detailed information on other risk factors including OGTT to define diabetes mellitus was available.
3. Validated questionnaires on alcohol consumption was used, however, still self-reported.
4. Finally, this was a cross sectional observational study and no conclusions in causality can be drawn. However, randomized controlled trials in this field are and will remain difficult to perform.

For peer review only

Introduction

Based on WHO from 2018 about 57% of adult population globally are alcohol consumers and 3 million deaths are attributed to alcohol consumption in 2016 (1, 2). A recently published meta-analysis could identify better health outcomes in people that had reported low to moderate alcohol consumption (3). On the other hand, several studies have shown that moderate levels of alcohol (i.e. alcohol equalling <1 drink per day (d/d) of alcohol in females and <2 d/d in males) have a protective effect on ischemic heart disease and chronic heart failure (CHF)(1, 4) while heavy episodic drinking negates from these effects (5). Interestingly, chronic excessive alcohol consumption is also reported to be associated with diastolic dysfunction (6), which is characterized by disturbed relaxation and increased preload. Diastolic dysfunction represents one out of three criteria for diastolic heart failure (7) and occurs often without impaired physical performance or other symptoms. The condition is common with prevalence in community-based studies varying from a few to 30 per cent (8, 9) and is associated with increase in risk to develop heart failure (10)

Although alcohol consumption is associated with common risk factors for diastolic dysfunction as hypertension(11) and obesity(12) the independent impact of low to medium-high levels of alcohol consumption on this condition in a community setting is still unclear. While there is consensus on deleterious effects of heavy drinking the role of low alcohol consumption on CVD and CHF risk and diastolic dysfunction is unknown. Also, the mechanisms of a protective effect in low doses and deleterious effect in higher doses haven't been explained yet.

Thus, the objective of this study was to explore the associations between alcohol intake and asymptomatic diastolic dysfunction with preserved systolic function in a population-based cohort.

For peer review only

Materials and Methods

Subjects

This study includes data from a surveillance of the population in Vara 2002–2003, a small community with 16 000 residents in a rural area of south-western Sweden(13). The sampling, inclusion of participants and UCG examination was conducted as previously described (14). Briefly, men (500) and women (538) 30 to 75 years (Mean age 55.4±13) were selected from the census register and invited to participate. Individuals below age 50 years were over-sampled three times as compared to those who were older. Participation was based on conducting an UCG and physical examination, filling in questionnaires and returning blood samples. Subjects with ejection fraction <45%, self-reported history of heart failure, arrhythmia, aortic insufficiency, or physical characteristics hindering echo were excluded from the analyses (N=20). The study profile is presented in figure 1.

Demographic and Lifestyle Data

Standardized physical examinations were performed and blood samples drawn including oral glucose tolerance tests (14). Diagnosis of hypertension was defined in accordance with international guidelines based on medical history of hypertension or three consecutive blood pressure measurements equal or higher than 140/90 mmHg (15). Diabetes was defined based on WHO recommendations (16). Information on previous cardiovascular disease (CVD) was collected through questionnaires. Information on smoking habits was also collected through questionnaires and participants were categorized as never smokers, former smokers, and current smokers.

Alcohol intake*:* Participants were asked to estimate the number of days they had been drinking alcohol the last month and the average amount of alcohol they had consumed during those days. Based on validated questionaries(17) for self-reported alcohol intake an estimation on gram per week of alcohol consumption was calculated for each participant. Based on quartiles of this estimated weekly alcohol consumption, the participants were divided into 3 groups. Non-drinkers were participants that reported no alcohol consumption or consumed alcohol only on a few occasions the last 12 months.

For peer review only - http://bmjopen.bmj.com/site/about/guidelines.xhtml

Highest quartile (>56 g/week) was defined as medium-high consumers and those with alcohol consumption ≤56g/week were defined as low consumers. One drink of alcohol was estimated equivalent to 15 grams of alcohol (18).

Level of leisure time physical activity (LTPA) was self-reported in four possible levels: *Sedentary LTPA* i.e. mostly physically inactive, *less strenuous LTPA* (walks, cycling, gardening etc.) <4 hours a week (h/w); *Medium LTPA* i.e. less strenuous LTPA ≥4h/w or *High LTPA* i.e. strenuous LTPA (jogging, swimming, tennis etc.) ≥2 h/w (19). Due to reduced number of individuals in the strenuous category we grouped group 3 and 4 together and defined it as reference.

Echocardiographic methods

All participants were examined by the same senior cardiologist, using echocardiography (General Electrics VingMed S 5 System, operating with a 3.5 MHz-probe) (14). Data was stored in the EchoPac System for playback analysis and measurement. Measurements for left ventricular calculations were taken from the Guidelines of the American Society of Echocardiography (20). A two-dimensional echocardiogram was formed from the left parasternal and apical windows for measurements. The EF was calculated from apical four and two chamber views with longitudinal systolic shortening mean index/ atrio-ventricular plane displacement technique in addition to a semi-quantitative visual estimate method (21, 22). The ventricular diastolic function was based on several parameters and their respective normal ranges were defined for different age categories (23). The E-wave-peak (early filling) to A-wave-peak (atrial filling), and the ratio were then determined. The isovolumetric relaxation time (IVRT), from the closure of the aortic valve to the opening of the mitral valve, was also measured. To indirectly measure distensibility in the left ventricle, the deceleration time (DT), the peak E to baseline-slope, was measured. Tissue Velocity Imaging (TVI) was used in both the septum and in the more stable lateral wall to detect pseudo-normalization. The patient was also told to perform the Valsalva manoeuvre during registration.

Diastolic dysfunction was defined according to the recommendations of the European Society of Cardiology(23, 24) when evidence of abnormal left ventricular diastolic relaxation, filling, diastolic distensibility and diastolic stiffness were found in the

For peer review only - http://bmjopen.bmj.com/site/about/guidelines.xhtml

presence of normal or mildly reduced left ventricular systolic function (EF≥45%). Diastolic dysfunction was categorized into three levels as: *impaired relaxation* i.e. early diastolic dysfunction; *pseudonormal* i.e. a more severe condition indicating increasing left ventricular filling pressures or *restrictive filling pattern*. Diastolic dysfunction was then further dichotomized as being normal or not with impaired relaxation, pseudonormal or restrictive filling patterns categorized as diastolic dysfunction. Another senior cardiologist – not otherwise involved in the project and unaware of the findings in the first examination – reviewed the diagnostic criteria and the echocardiographic methods and re-examined a random sample (n=30) of the study sample using stored images. The results were consistent showing identical categorization.

Left ventricular mass was defined as: LVM = 0.8 × (1.04 [(diastolic dimension + posterior wall thickness + septal thickness)3 − (diastolic dimension)3]) + 0.6 g. LVM was then indexed for height2.7(LVM-index) [16], and LVH was defined as LVM-index ≥47.3 g/m 2.7 based on guidelines (25).

Statistical analysis

Standard methods were used for descriptive statistics which were presented in terms of mean and standard deviation for all continuous variables and counts with percentage of categorical variables. All the statistical analysis were performed using program R 4.1.2. Possible confounding was addressed by stratification and multivariate analyses. Theoretical multiple logistic regressions were used to evaluate the association between diastolic dysfunction and alcohol consumption categories, with adjustments for differences in age, sex, smoker, physical activity, diabetes, and hypertension. These associations were expressed as odds ratios (OR) with 95% confidence intervals (CI). All tests were 2-sided and statistical significance were assumed at p <0.05.

Ethical considerations

The investigation conforms to the principles outlined in the Declaration of Helsinki. The research ethics committee at the University of Gothenburg approved the study (DNR Ö199-01). The participants provided written informed consent to their participation in the health survey, and a further written informed consent was obtained before undergoing an ultrasound-Doppler examination of the heart.

For peer review only - http://bmjopen.bmj.com/site/about/guidelines.xhtml

Results

In total, 79 of 500 men (16.5%) and 58 of 538 (12.9%) women were diagnosed with diastolic dysfunction (*p*=0.037). Totally 94% of the cases were categorized as *impaired relaxation* (74 men, 55 women) and 6% as *pseudonormal* (5 men, 3 women). There were no cases of severe *restrictive* filling patterns.

Alcohol consumption was common in both genders; men 84.5% (412/486) and women 68.8% (375/527), however, significant differences were observed between men and women with women being non-drinkers to a larger extent (p<0.001). In this observational study, non-drinkers were older, had higher blood pressure and had more often diabetes, however, the prevalence of diastolic dysfunction was similar in all three groups (p=0.500). Meanwhile, among subjects not using alcohol a higher rate of left ventricular hypertrophy (LVH) was observed. Characteristics of the study population as well as echocardiographic characteristics related to level of alcohol intake are presented in *Table 1*.

In a multivariable theoretical model, adjusted for possible confounders and mediators in the association between alcohol consumption and diastolic dysfunction, we found that alcohol consumption was significantly associated with increased risk of diastolic dysfunction. Interestingly, even low alcohol consumption was strongly associated with increased risk for diastolic dysfunction (OR=2.2 95% CI 1.3-3.9). The estimates were even higher for participants with moderate to high alcohol consumption (OR=3.1 95% CI 1.6-6.2) with a strong significant trend (p<0.001). No significant differences in these associations were observed between men and women (p for interaction >0.25). High age, male sex, hypertension, high BMI, and a low level of leisure time physical activity were all also independently associated with diastolic dysfunction (*Table 2).*

Similar analyses were computed using LVH as outcome, but no significant association was found with alcohol consumption (*Table3*).

For peer review only - http://bmjopen.bmj.com/site/about/guidelines.xhtml

Discussion

In this cross-sectional observational study, we found a strong and independent association between self-reported alcohol consumption and diastolic dysfunction. Interestingly, our data show an increased risk of having diastolic dysfunction already in subjects with low-level of alcohol consumption. Moreover, in gender specific analyses no gender differences were observed suggesting a similar effect of alcohol in both men and women. Despite these findings, no significant association between alcohol intake and LVH were observed. Like previous observations, age, gender, hypertension, leisure time physically activity levels, and BMI were all strongly and independently associated with diastolic dysfunction.

These findings are not in line with most of the observations on the role of alcohol in heart failure. In fact, population-based studies have shown that *moderate* levels of alcohol consumption (i.e. <1 drinks per day (d/d) in females and <2 d/d in males) have a protective effect on ischemic heart disease and decreases the incidence of CHF (1, 26) (3). The beneficial effect of a low to moderate amount of alcoholic intake are not reported by all epidemiological studies (27, 28). A 'healthy cohort effect' is reported as one plausible explanation to the favourable effects of moderate consumption (27, 28) where the confounding effects of for example physical activity, mental health, psychosocial, socioeconomic and social network related factors seldom are accounted for (28). Moreover, in a recent observation in HUNT study (29) no protective effect of low-alcohol consumption on left ventricular function was observed, however, a positive linear association was observed between alcohol consumption and left ventricular mass and end diastolic diameter. The findings of HUNT-study are in line with our findings however the significance in the association was lost in their multivariable analyses. Interestingly, before the adjustments in our cohort higher prevalence of diastolic dysfunction was observed in nondrinkers. These differences were not significant. The same group had also higher age, lower level of physical activities, higher systolic blood pressure and higher prevalence of hypertension and diabetes. It was first after the adjustments for these factors that a positive linear association between alcohol consumption and diastolic dysfunction was observed. These findings suggest no protective effect of alcohol at low

For peer review only - http://bmjopen.bmj.com/site/about/guidelines.xhtml

levels and a possible reversal causality. Also, alcohol seems not to be related with better diastolic dysfunction, but healthy and younger subjects are more prone to drink alcohol and they have of course better diastolic function but not of alcohol consumption.

Several alcohol metabolites are identified as particularly toxic to the myocardium: ethanol, acetaldehyde (the metabolic product of alcohol) and fatty acid ethyl esters (30, 31). Alcohol also impairs the transport of calcium ions from the sarcoplasma to the sarcoplasmatic reticulum (32, 33) resulting in diastolic dysfunction characterized by delayed myocardial relaxation (34) and impaired left ventricular (LV) diastolic filling. Moderate versus high doses of alcohol affect myocardial function differently through oxidative stress, haemostatic factors, levels of C-reactive protein, interleukins, and TNF alpha (27).

There have been observations that suggest that patterns of drinking and food intake might explain at least partially the damages of alcohol in myocardium(5, 35, 36). In fact, the Mediterranean countries with a low prevalence of CVD, alcohol is commonly consumed with meals as compared to Russia with a high prevalence of CVD where binge drinking is much more frequent (28). However, some studies have shown there is evidence that alcoholic cardiomyopathy can be developed even in absence of malnutrition(37) while these deleterious effects are exaggerated of malnutrition(38, 39).

Interestingly, our results did not show any significant association with LVH. One reason of the lack of association might be the relatively low number of individuals with high consumption in this cohort. Measurable structural remodelling might need higher level of alcohol consumption. In fact, a large population study in South Korea with significantly larger number of participants compared with our study, could find a strong association between alcohol consumption and LVH(40).

Strengths and limitations

Echocardiography was used in this large population-based cohort using standardized age-dependent criteria to identify diastolic disfunction based on recommended by international expert groups. The re-evaluation of a sub-sample by a senior cardiologist confirmed the first assessment and the consistency in assessment of prevalence of diastolic dysfunction and in identified risk factors between our findings and other

For peer review only - http://bmjopen.bmj.com/site/about/guidelines.xhtml

comprehensive surveys using Doppler techniques, lend further support to the validity of our data.

The characteristics of alcohol consumers and prevalence of alcohol consumption was in accordance with other comparable community-based surveys (41, 42). This, however, might be a source of underestimation of the alcohol consumption. In fact, in several studies there is an underestimation of self-reported alcohol consumption(43) and there is need of more objective and reliable measures of alcohol consumption. However, the underestimation should be more frequent and mostly in the group with higher consumption. This would not have an impact effect on our results in the association found between low alcohol consumption and diastolic dysfunction. Finally, this was a cross sectional observation and no conclusions in causality can be drawn.

Conclusion

Our study revealed a significant association between alcohol consumption and diastolic dysfunction starting already at low levels that was supported by a dose-dependent pattern. More prospective surveys with adequate measurement of alcohol consumption and possible confounders are needed for a better understanding of the underlying causality and mechanisms. These results are important on the public health policies and information on alcohol use.

Competing interests

The authors declare that they have no competing interests.

Acknowledgements and funding

We are indebted to all participants in the Vara Cohort. Without them this study would not been possible. The Knut and Alice Wallenberg foundation is recognized for generous support. This work was supported by: The Swedish Science Council; Sahlgrenska Academy, University of Gothenburg; Skaraborg Institute; The Health & Medical Care Committee of the Regional Executive Board of the Region Västragötalands region VGFOUREG-86294, VGFOUREG-969459, ALFGBG-966255, ALFGBG-971147; Skaraborg Primary Care; Skane Region; Lund University; University Hospital of Malmoe donation Foundation; Påhlsson Foundation; Crafoord Foundation; Lundberg Foundation,

For peer review only

the Swedish Heart and Lung foundation and the Wallenberg Center for Molecular Medicine, Lund University; and Lund University. The funding source was not involved in the work with the article.

Author contribution: All authors read and approved the final manuscript. Research idea and study design: LB, BD, UL, MH; Data acquisition: UL, LR, BD, MH; Data analysis and interpretation: LB, UL, BD, MM, MH; Statistical analyses: LB, BD, YL, UL. Each author contributed important intellectual content during manuscript drafting or revision, accepts personal accountability for the author's own contributions, and agrees to ensure that questions pertaining to the accuracy or integrity of any portion of the work are appropriately investigated and resolved.

For peer review only

For peer review only - http://bmjopen.bmj.com/site/about/guidelines.xhtml

References

1.Djousse, L. and Gaziano, J.M. (2007). Alcohol consumption and risk of heart failure in the Physicians' Health Study I. Circulation 115: 34-39.
2.World Health Organization. Management of Substance Abuse Team. Global status report on alcohol and health, pvolumes, World Health Organization, Geneva, Switzerland.
3.Zhong, L., Chen, W., Wang, T., Zeng, Q., Lai, L., Lai, J., Lin, J. and Tang, S. (2022). Alcohol and Health Outcomes: An Umbrella Review of Meta-Analyses Base on Prospective Cohort Studies. Front Public Health 10: 859947.
4.Larsson, S.C., Wallin, A. and Wolk, A. (2018). Alcohol consumption and risk of heart failure: Meta-analysis of 13 prospective studies. Clin Nutr 37: 1247-1251.
5.Murray, R.P., Connett, J.E., Tyas, S.L., Bond, R., Ekuma, O., Silversides, C.K. and Barnes, G.E. (2002). Alcohol volume, drinking pattern, and cardiovascular disease morbidity and mortality: is there a U-shaped function? Am J Epidemiol 155: 242-248.
6.Lazarevic, A.M., Nakatani, S., Neskovic, A.N., Marinkovic, J., Yasumura, Y., Stojicic, D., Miyatake, K., Bojic, M. and Popovic, A.D. (2000). Early changes in left ventricular function in chronic asymptomatic alcoholics: relation to the duration of heavy drinking. J Am Coll Cardiol 35: 1599-1606.
7.(1998). How to diagnose diastolic heart failure. European Study Group on Diastolic Heart Failure. Eur Heart J 19: 990-1003.
8.Fischer, M., Baessler, A., Hense, H.W., Hengstenberg, C., Muscholl, M., Holmer, S., Doring, A., Broeckel, U., Riegger, G. and Schunkert, H. (2003). Prevalence of left ventricular diastolic dysfunction in the community. Results from a Doppler echocardiographic-based survey of a population sample. Eur Heart J 24: 320-328.
9.Redfield, M.M., Jacobsen, S.J., Burnett, J.C., Jr., Mahoney, D.W., Bailey, K.R. and Rodeheffer, R.J. (2003). Burden of systolic and diastolic ventricular dysfunction in the community: appreciating the scope of the heart failure epidemic. JAMA 289: 194-202.
10.Vogel, M.W., Slusser, J.P., Hodge, D.O. and Chen, H.H. (2012). The natural history of preclinical diastolic dysfunction: a population-based study. Circ Heart Fail 5: 144-151.
11.Liu, F., Liu, Y., Sun, X., Yin, Z., Li, H., Deng, K., Zhao, Y., Wang, B., Ren, Y., Liu, X., Zhang, D., Chen, X., Cheng, C., Liu, L., Liu, D., Chen, G., Hong, S., Wang, C., Zhang, M. and Hu, D. (2020). Race- and sex-specific association between alcohol consumption and hypertension in 22 cohort studies: A systematic review and meta-analysis. Nutr Metab Cardiovasc Dis 30: 1249-1259.
12.Chakraborty, S. (2014). Analysis of NHANES 1999-2002 data reveals noteworthy association of alcohol consumption with obesity. Ann Gastroenterol 27: 250-257.
13.Larsson, C.A., Kroll, L., Bennet, L., Gullberg, B., Rastam, L. and Lindblad, U. (2012). Leisure time and occupational physical activity in relation to obesity and insulin resistance: a population-based study from the Skaraborg Project in Sweden. Metabolism 61: 590-598.
14.Ingelsson, E., Bennet, L., Ridderstrale, M., Soderstrom, M., Rastam, L. and Lindblad, U. (2008). The PPARGC1A Gly482Ser polymorphism is associated with left ventricular diastolic dysfunction in men. BMC Cardiovasc Disord 8: 37.

For peer review only

15.(1999). 1999 World Health Organization-International Society of Hypertension Guidelines for the Management of Hypertension. Guidelines Subcommittee. J Hypertens 17: 151-183.
16.Alberti, K.G. and Zimmet, P.Z. (1998). Definition, diagnosis and classification of diabetes mellitus and its complications. Part 1: diagnosis and classification of diabetes mellitus provisional report of a WHO consultation. Diabet Med 15: 539-553.
17.Goransson, M. and Hanson, B.S. (1994). How much can data on days with heavy drinking decrease the underestimation of true alcohol consumption? J Stud Alcohol 55: 695-700.
18.Bantle, J.P., Wylie-Rosett, J., Albright, A.L., Apovian, C.M., Clark, N.G., Franz, M.J., Hoogwerf, B.J., Lichtenstein, A.H., Mayer-Davis, E., Mooradian, A.D. and Wheeler, M.L. (2006). Nutrition recommendations and interventions for diabetes--2006: a position statement of the American Diabetes Association. Diabetes Care 29: 2140-2157.
19.Nyholm, M., Gullberg, B., Haglund, B., Rastam, L. and Lindblad, U. (2008). Higher education and more physical activity limit the development of obesity in a Swedish rural population. The Skaraborg Project. Int J Obes (Lond) 32: 533-540.
20.Lang, R.M., Bierig, M., Devereux, R.B., Flachskampf, F.A., Foster, E., Pellikka, P.A., Picard, M.H., Roman, M.J., Seward, J., Shanewise, J.S., Solomon, S.D., Spencer, K.T., Sutton, M.S. and Stewart, W.J. (2005). Recommendations for chamber quantification: a report from the American Society of Echocardiography's Guidelines and Standards Committee and the Chamber Quantification Writing Group, developed in conjunction with the European Association of Echocardiography, a branch of the European Society of Cardiology. J Am Soc Echocardiogr 18: 1440-1463.
21.Jarnert, C., Mejhert, M., Ring, M., Persson, H. and Edner, M. (2000). Doppler tissue imaging in congestive heart failure patients due to diastolic or systolic dysfunction: a comparison with Doppler echocardiography and the atrio-ventricular plane displacement technique. Eur J Heart Fail 2: 151-160.
22.Gudmundsson, P., Rydberg, E., Winter, R. and Willenheimer, R. (2005). Visually estimated left ventricular ejection fraction by echocardiography is closely correlated with formal quantitative methods. Int J Cardiol 101: 209-212.
23.Oh, J.K., Appleton, C.P., Hatle, L.K., Nishimura, R.A., Seward, J.B. and Tajik, A.J. (1997). The noninvasive assessment of left ventricular diastolic function with two-dimensional and Doppler echocardiography. J Am Soc Echocardiogr 10: 246-270.
24.Swedberg, K., Cleland, J., Dargie, H., Drexler, H., Follath, F., Komajda, M., Tavazzi, L., Smiseth, O.A., Gavazzi, A., Haverich, A., Hoes, A., Jaarsma, T., Korewicki, J., Levy, S., Linde, C., Lopez-Sendon, J.L., Nieminen, M.S., Pierard, L. and Remme, W.J. (2005). Guidelines for the diagnosis and treatment of chronic heart failure: executive summary (update 2005): The Task Force for the Diagnosis and Treatment of Chronic Heart Failure of the European Society of Cardiology. Eur Heart J 26: 1115-1140.
25.de Simone, G., Devereux, R.B., Chinali, M., Roman, M.J., Lee, E.T., Resnick, H.E. and Howard, B.V. (2009). Metabolic syndrome and left ventricular hypertrophy in the prediction of cardiovascular events: the Strong Heart Study. Nutr Metab Cardiovasc Dis 19: 98-104.
26.Walsh, C.R., Larson, M.G., Evans, J.C., Djousse, L., Ellison, R.C., Vasan, R.S. and Levy, D. (2002). Alcohol consumption and risk for congestive heart failure in the Framingham Heart Study. Ann Intern Med 136: 181-191.

For peer review only - http://bmjopen.bmj.com/site/about/guidelines.xhtml

27.Bau, P.F., Bau, C.H., Rosito, G.A., Manfroi, W.C. and Fuchs, F.D. (2007). Alcohol consumption, cardiovascular health, and endothelial function markers. Alcohol 41: 479-488.
28.Fuchs, F.D. and Chambless, L.E. (2007). Is the cardioprotective effect of alcohol real? Alcohol 41: 399-402.
29.Gemes, K., Janszky, I., Strand, L.B., Laszlo, K.D., Ahnve, S., Vatten, L.J., Dalen, H. and Mukamal, K.J. (2018). Light-moderate alcohol consumption and left ventricular function among healthy, middle-aged adults: the HUNT study. BMJ Open 8: e020777.
30.Beckemeier, M.E. and Bora, P.S. (1998). Fatty acid ethyl esters: potentially toxic products of myocardial ethanol metabolism. J Mol Cell Cardiol 30: 2487-2494.
31.Ren, J. and Wold, L.E. (2008). Mechanisms of alcoholic heart disease. Ther Adv Cardiovasc Dis 2: 497-506.
32.Cofan, M., Nicolas, J.M., Fernandez-Sola, J., Robert, J., Tobias, E., Sacanella, E., Estruch, R. and Urbano-Marquez, A. (2000). Acute ethanol treatment decreases intracellular calcium-ion transients in mouse single skeletal muscle fibres in vitro. Alcohol Alcohol 35: 134-138.
33.Noren, G.R., Staley, N.A., Einzig, S., Mikell, F.L. and Asinger, R.W. (1983). Alcohol-induced congestive cardiomyopathy: an animal model. Cardiovasc Res 17: 81-87.
34.Kupari, M., Koskinen, P., Suokas, A. and Ventila, M. (1990). Left ventricular filling impairment in asymptomatic chronic alcoholics. Am J Cardiol 66: 1473-1477.
35.Urquiaga, I., Guasch, V., Marshall, G., San Martin, A., Castillo, O., Rozowski, J. and Leighton, F. (2004). Effect of Mediterranean and Occidental diets, and red wine, on plasma fatty acids in humans. An intervention study. Biol Res 37: 253-261.
36.Britton, A. and McKee, M. (2000). The relation between alcohol and cardiovascular disease in Eastern Europe: explaining the paradox. J Epidemiol Community Health 54: 328-332.
37.Urbano-Marquez, A., Estruch, R., Navarro-Lopez, F., Grau, J.M., Mont, L. and Rubin, E. (1989). The effects of alcoholism on skeletal and cardiac muscle. N Engl J Med 320: 409-415.
38.Fernandez-Sola, J. (2015). Cardiovascular risks and benefits of moderate and heavy alcohol consumption. Nat Rev Cardiol 12: 576-587.
39.Piano, M.R. and Phillips, S.A. (2014). Alcoholic cardiomyopathy: pathophysiologic insights. Cardiovasc Toxicol 14: 291-308.
40.Park, S.K., Moon, K., Ryoo, J.H., Oh, C.M., Choi, J.M., Kang, J.G., Chung, J.Y. and Young Jung, J. (2018). The association between alcohol consumption and left ventricular diastolic function and geometry change in general Korean population. Eur Heart J Cardiovasc Imaging 19: 271-278.
41.Bostrom, G. and Nykvist, K. (2004). Lifestyle and health (in Swedish), The National Institute of Public Health in Sweden. Available at *http://www.fhi.se/upload/ar2005/rapporter/r200448levnadsvanorhalsa0504.pdf*
42.Wilsgaard, T. and Jacobsen, B.K. (2007). Lifestyle factors and incident metabolic syndrome. The Tromso Study 1979-2001. Diabetes Res Clin Pract 78: 217-224.
43.Stockwell, T., Zhao, J., Sherk, A., Rehm, J., Shield, K. and Naimi, T. (2018). Underestimation of alcohol consumption in cohort studies and implications for alcohol's contribution to the global burden of disease. Addiction 113: 2245-2249.

For peer review only - http://bmjopen.bmj.com/site/about/guidelines.xhtml

For peer review only

For peer review only - http://bmjopen.bmj.com/site/about/guidelines.xhtml

For peer review only

For peer review only - http://bmjopen.bmj.com/site/about/guidelines.xhtml

Figure 1. Study population in the Vara Cohort 2002-2003

Commented [HBL1]: Lägg till när undersökning genomförd

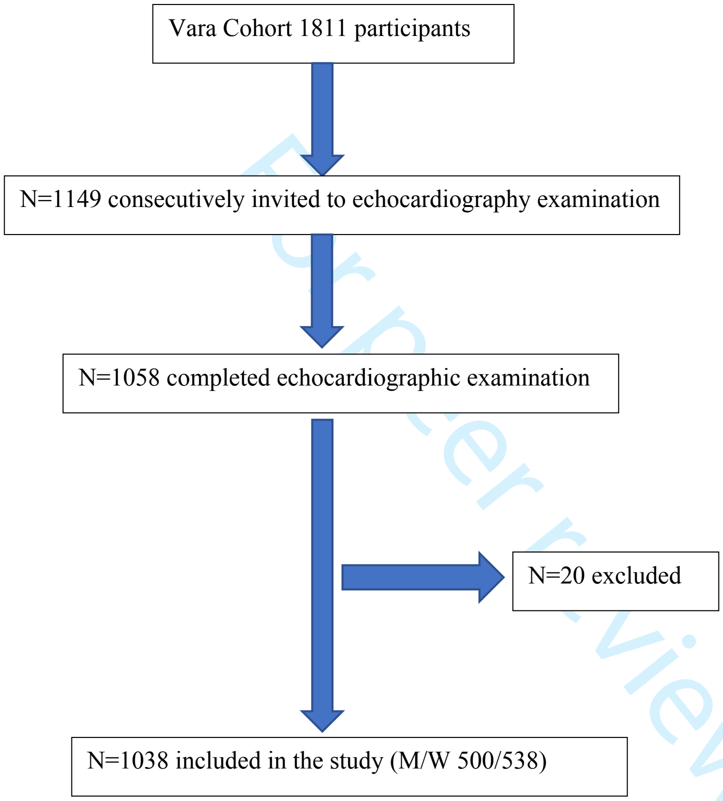

*Cases excluded due to at least one of the following reasons: arrhythmia/atrial fibrillation n=8, physical characteristics hindering UCG n=6, self-reported history of heart failure n=6, EF<45% n=2 and/or aortic insufficiency n=2.

Table 1 Characteristics of participants related to level of alcohol consumption.

Variables	alcohol consumption Non consumers n=226	Low n=531	Medium-High n=254	P*
Diastolic dysfunction[a]	35(15.5%)	66 (12.4%)	32 (12.6%)	0.500
Age (years)[b]	55.4(12.8)	49.5(11.5)	48 (10.7)	0.050
Women[a]	152 (67.3%)	289 (54.4%)	85 (33.5%)	<0.001
BMI (kg/m2)[b]	27.8(5.4)	26.8(4.2)	27 (3.9)	<0.001
SBP (mmHg)[b]	128.4(18.9)	123.6(17.1)	123.2 (15.6)	0.001
DBP (mmHg)[b]	71.1 (9.7)	71.1 (10.3)	72.1 (10.1)	0.300
HDL (mmol/L)[b]	1.2(0.3)	1.3(0.3)	1.3 (0.3)	0.100
TG (g/L)[b]	1.4(0.7)	1.3(0.8)	1.3 (0.7)	0.700
Level of physical activities				0.020
mobile	57(25.2%)	191(36.0%)	90(35.4%)	
medium	147(65%)	312(58.8%)	149(58.7%)	
sedentary	22(9.7%)	28(5.3%)	12(5.9%)	
Hypertension[a]	61 (27.0%)	93 (17.5%)	33 (13%)	<0.001
Diabetes[a]	29 (12.8%)	34 (6.4%)	10 (3.9%)	<0.001
smoker[a]	39 (17.3%)	92 (17.3%)	54 (21.4%)	0.300
E/A ratio	1.2(0.4)	1.4(0.4)	1.4(0.4)	<0.001
IVRT	98.4(21.9)	91.6(20.1)	93.2(21.2)	<0.001
EJECTION FRACTION	72.2(9.6)	74.4(8.4)	74.4(7.8)	0.005
SEPT_wall	1(0.2)	0.9(0.2)	0.9(0.2)	0.008
POST_wall	1(0.3)	0.9(0.2)	1(0.5)	0.010
LAD	3.3(0.5)	3.2(0.5)	3.3(0.5)	0.040
LV-mass (Median and 5th, 95th percentiles)	37.8 (23.4, 62)	34.5 (23.8, 55.6)	35.1 (23.9, 57.5)	0.050*
LVH	52 (23.0%)	70(13.2%)	32(12.6%)	0.001#

[a] data is presented as counts (percent).[b] data are presented with mean (standard deviation)

*P values are based ANOVA if the variables are continuous or based on chi-square test if the variables are binary. Medium-high is defined as consumption >56 g/week and low those with alcohol consumption ≤56g/week.

For peer review only

Table 2: Association between risk factors and diastolic dysfunction

	OR	95% confidence interval
Age	1.14	(1.11-1.18)
Men vs Women	1.63	(1.02-2.63)
BMI	1.07	(1.01-1.11)
Hypertension	2.68	(1.65-4.33)
Type 2 Diabetes	1.88	(1.00-3.53)
Current smoking	1.76	(0.96-3.15)
Leisure time physical activity		
medium vs mobile	1.28	(0.75-2.23)
sedentary vs mobile	2.82	(1.15-6.84)
Alcohol consumption		
None	1	ref
low vs none	2.25	(1.29-4.02)
high vs none	3.06	(1.55-6.15)

Multivariable logistic regression analyses are used to investigate the association between explanatory variables and diastolic dysfunction. OR Odds ratio, BMI body mass index.

For peer review only

Table 3: Association between risk factors and left ventricular hypertrophy		
	OR	confidence interval
Age	1.08	(1.06-1.1)
Men vs Women	1.16	(0.75-1.78)
BMI	1.16	(1.11-1.22)
Hypertension	3.20	(2.05-5.01)
Type 2 Diabetes	1.30	(0.70-2.38)
Current smoking	1.53	(0.89-2.57)
Leisure time physical activity		
medium vs mobile	0.96	(0.60-1.56)
sedentary vs mobile	1.18	(0.50-2.69)
Alcohol consumption		
Non drinkers	1	Ref
low vs non	1.08	(0.59-1.98)
high vs non	1.53	(0.89-2.57)

Multivariable logistic regression analyses are used to investigate the association between explanatory variables and left ventricular hypertrophy. OR Odds ratio, BMI body mass index

For peer review only

BMJ Open

Association between self-reported alcohol consumption and diastolic dysfunction, a cross-sectional study

Journal:	*BMJ Open*
Manuscript ID	bmjopen-2022-069937.R1
Article Type:	Original research
Date Submitted by the Author:	02-Jun-2023
Complete List of Authors:	Daka, Bledar; University of Gothenburg Sahlgrenska Academy, Medicine<br>Bennet, Louise; Lund University, Family Medicine, Deaprtment of Clinical Sciences<br>Råstam, Lennart ; Lund University, Department of Clinical Sciences, Malmö, Sweden<br>Hellgren, Margareta; University of Gothenburg<br>Li, Ying; Gothenburg University Library<br>Magnusson, Martin; Lund University<br>Lindblad, Ulf; Lund University, Department of Clinical Sciences in Malmö
**Primary Subject Heading**:	Cardiovascular medicine
Secondary Subject Heading:	General practice / Family practice, Epidemiology
Keywords:	Echocardiography < CARDIOLOGY, PREVENTIVE MEDICINE, Heart failure < CARDIOLOGY

SCHOLARONE™
Manuscripts

I, the Submitting Author has the right to grant and does grant on behalf of all authors of the Work (as defined in the below author licence), an exclusive licence and/or a non-exclusive licence for contributions from authors who are: i) UK Crown employees; ii) where BMJ has agreed a CC-BY licence shall apply, and/or iii) in accordance with the terms applicable for US Federal Government officers or employees acting as part of their official duties; on a worldwide, perpetual, irrevocable, royalty-free basis to BMJ Publishing Group Ltd ("BMJ") its licensees and where the relevant Journal is co-owned by BMJ to the co-owners of the Journal, to publish the Work in this journal and any other BMJ products and to exploit all rights, as set out in our licence.

The Submitting Author accepts and understands that any supply made under these terms is made by BMJ to the Submitting Author unless you are acting as an employee on behalf of your employer or a postgraduate student of an affiliated institution which is paying any applicable article publishing charge ("APC") for Open Access articles. Where the Submitting Author wishes to make the Work available on an Open Access basis (and intends to pay the relevant APC), the terms of reuse of such Open Access shall be governed by a Creative Commons licence – details of these licences and which Creative Commons licence will apply to this Work are set out in our licence referred to above.

Other than as permitted in any relevant BMJ Author's Self Archiving Policies, I confirm this Work has not been accepted for publication elsewhere, is not being considered for publication elsewhere and does not duplicate material already published. I confirm all authors consent to publication of this Work and authorise the granting of this licence.

For peer review only

Association between self-reported alcohol consumption and diastolic dysfunction, a cross-sectional study

Bledar Daka MD MD PhD[1], Louise Bennet MD, PhD[2, 3], Lennart Råstam MD, PhD[2], Margareta I Hellgren MD PhD [1, 5], Ying Li PhD[1], Martin Magnusson MD PhD[2,6, 7, 8], Ulf Lindblad MD, PhD[1, 5]

[1] Family medicine, School of Public Health and Community Medicine, The Sahlgrenska Academy, the University of Gothenburg, Sweden
[2] Lund University, Department of Clinical Sciences, Malmö, Sweden
[3] Clinical Trial Unit, Skåne University Hospital, Lund, Sweden
[4] Department of Cardiology, Skaraborg Hospital, Skövde, Sweden
[5] Skaraborg Institute, Skövde, Sweden
[6] Department of Cardiology, Skåne University Hospital, Malmö, Sweden
[7] Hypertension in Africa Research Team (HART); North-West University, Potchefstroom, South Africa
[8] Wallenberg Center for Molecular Medicine, Lund University, Lund, Sweden

Correspondence to: Bledar Daka Associated Professor, School of Public Health and Community Medicine/Primary Health Care, Hälsovetarbacken 7, Box 454, SE 405 30 Göteborg, Sweden
Phone: +46 31 786 68 31
e-mail: bledar.daka@allmed.gu.se

For peer review only

Abstract

Background and objectives: While alcohol consumption is associated with common risk factors for diastolic dysfunction the independent impact of low levels of alcohol consumption on this condition in a community setting is still unclear.

Thus, the aim of this study was to explore this association in a representative population sample employing optimal echocardiographic techniques.

Design: Cross-sectional observational study in community-based population

Settings, participants, and methods: Men (500) and women (538) 30 to 75 years (Mean age 55.4±13) were consecutively invited to a physical exam, interview, conventional echocardiography, including Tissue Velocity Imaging. Diastolic dysfunction was defined according to the European Society of Cardiology criteria, excluding subjects with ejection fraction <45%, self-reported history of heart failure, or atrial fibrillation on ECG. Self-reported alcohol intake using a validated questionnaire was categorized as *no intake, low and medium-high* intake.

Results: In total, 500 men and 538 women were successfully examined. Diastolic dysfunction was identified in 16% (79/500) of the men and 13% (58/538) of the women, p=0.037. The multivariable adjusted model revealed a strong and independent association between alcohol intake and diastolic dysfunction. In fact, using no alcohol intake as reference, diastolic dysfunction was independently associated with alcohol consumption in a dose-dependent fashion; *low consumption,* OR 2.3 (95% CI 1.3 to 4.0) and *medium-high consumption OR* 3.1 (95% CI 1.6 to 6.2), respectively.

Conclusion: There was a significant association between alcohol consumption and diastolic dysfunction starting already at low levels that was supported by a dose-dependent pattern. These results need confirmatory studies and are important in public health policies.

Keywords: *diastolic dysfunction, echocardiography, heart failure, alcohol consumption*, public health

For peer review only - http://bmjopen.bmj.com/site/about/guidelines.xhtml

Strengths and limitations of the study

1. Echocardiography was performed in a large population-based cohort using standardized methods. The re-evaluation of a sub-sample by a senior cardiologist confirmed the first assessment and the consistency.
2. Detailed information on other risk factors including oral glucose tolerance test to define diabetes mellitus was available.
3. Validated questionnaires on alcohol consumption was used, however, still self-reported.
4. Finally, this was a cross sectional observational study and no conclusions in causality can be drawn. However, randomized controlled trials in this field are and will remain difficult to perform.

For peer review only

Introduction

Based on World Health Organisation from 2018 about 57% of adult population globally are alcohol consumers and 3 million deaths are attributed to alcohol consumption in 2016 (1, 2). A recently published meta-analysis could identify better health outcomes in people that had reported low to moderate alcohol consumption (3). On the other hand, several studies have shown that moderate levels of alcohol (i.e. alcohol equalling <1 drink per day (d/d) of alcohol in females and <2 d/d in males) have a protective effect on ischemic heart disease and chronic heart failure (CHF)(1, 4) while heavy episodic drinking negates from these effects (5). Interestingly, chronic excessive alcohol consumption is also reported to be associated with diastolic dysfunction (6), which is characterized by disturbed relaxation and increased preload. Diastolic dysfunction represents one out of three criteria for diastolic heart failure (7) and occurs often without impaired physical performance or other symptoms. The condition is common with prevalence in community-based studies varying from a few to 30 per cent (8, 9) and is associated with increase in risk to develop heart failure (10)

In patients with hypertension moderate alcohol consumption have been associated with diastolic dysfunction (11). Although alcohol consumption is associated with common risk factors for diastolic dysfunction as hypertension(12) and obesity(13) the independent impact of low to levels of alcohol consumption on this condition in a community setting is still unclear. While there is consensus on deleterious effects of heavy drinking the role of low alcohol consumption on CVD and CHF risk and diastolic dysfunction is unknown. Also, the mechanisms of a protective effect in low doses and deleterious effect in higher doses haven't been explained yet.

Thus, the objective of this study was to explore the associations between alcohol intake and asymptomatic diastolic dysfunction with preserved systolic function in a population-based cohort.

For peer review only - http://bmjopen.bmj.com/site/about/guidelines.xhtml

Materials and Methods

Subjects

This study includes data from a surveillance of the population in Vara 2002–2003, a small community with 16 000 residents in a rural area of south-western Sweden(14). The sampling, inclusion of participants and echocardiography-examination was conducted as previously described (15). Briefly, men (500) and women (538) 30 to 75 years were selected from the census register and invited to participate. Individuals below age 50 years were over-sampled three times as compared to those who were older. Participation was based on conducting an echocardiography and physical examination, filling in questionnaires and returning blood samples. Subjects with ejection fraction <45%, self-reported history of heart failure, arrhythmia, aortic insufficiency, or physical characteristics hindering echo were excluded from the analyses (N=20). The study profile is presented in Figure 1

Figure 1

.

Demographic and Lifestyle Data

Standardized physical examinations were performed and blood samples drawn including oral glucose tolerance tests (15). Blood pressure was measured in lying position after 5 minutes rest. Two measurements were done with 1 minute apart and the mean value was used. Diagnosis of hypertension was defined in accordance with international guidelines based on medical history of hypertension or three consecutive blood pressure measurements equal or higher than 140/90 mmHg (16). Diabetes was defined based on World Health Organisation recommendations (17). Information on previous cardiovascular disease (CVD) was collected through questionnaires. Information on smoking habits was also collected through questionnaires and participants were categorized as never smokers, former smokers, and current smokers.

Alcohol intake*:* Participants were asked to estimate the number of days they had been drinking alcohol the last month and the average amount of alcohol they had consumed during those days. Based on validated questionaries(18) for self-reported alcohol intake

For peer review only - http://bmjopen.bmj.com/site/about/guidelines.xhtml

an estimation on gram per week of alcohol consumption was calculated for each participant. Based on quartiles of this estimated weekly alcohol consumption, the participants were divided into 3 groups. Non-drinkers were participants that reported no alcohol consumption the last month. Highest quartile (>56 g/week) was defined as medium-high consumers and those with alcohol consumption ≤56g/week were defined as low consumers. One drink of alcohol was estimated equivalent to 15 grams of alcohol (19).

Level of leisure time physical activity (LTPA) was self-reported in four possible levels: *Sedentary LTPA* i.e. mostly physically inactive, *less strenuous LTPA* (walks, cycling, gardening etc.) <4 hours a week (h/w); *Medium LTPA* i.e. less strenuous LTPA ≥4h/w or *High LTPA* i.e. strenuous LTPA (jogging, swimming, tennis etc.) ≥2 h/w (20). Due to reduced number of individuals in the strenuous category we grouped group 3 and 4 together and defined it as reference.

Echocardiographic methods

All participants were examined by the same senior cardiologist, using echocardiography (General Electrics VingMed S 5 System, operating with a 3.5 MHz-probe) (15). The same cardiologist performed all the measurements and had no access on information on alcohol drinking habits. Data was stored in the EchoPac System for playback analysis and measurement. Measurements for left ventricular calculations were taken from the Guidelines of the American Society of Echocardiography (21). A two-dimensional echocardiogram was formed from the left parasternal and apical windows for measurements. The EF was calculated from apical four and two chamber views with longitudinal systolic shortening mean index/ atrio-ventricular plane displacement technique in addition to a semi-quantitative visual estimate method (22, 23). The ventricular diastolic function was based on several parameters and their respective normal ranges were defined for different age categories (24). The E-wave-peak (early filling) to A-wave-peak (atrial filling), and the ratio were then determined. The isovolumetric relaxation time (IVRT), from the closure of the aortic valve to the opening of the mitral valve, was also measured. To indirectly measure distensibility in the left ventricle, the deceleration time (DT), the peak E to baseline-slope, was measured. Tissue

For peer review only

Velocity Imaging (TVI) was used in both the septum and in the more stable lateral wall to detect pseudo-normalization. The patient was also told to perform the Valsalva manoeuvre during registration.

Diastolic dysfunction was defined according to the recommendations of the European Society of Cardiology(24, 25) when evidence of abnormal left ventricular diastolic relaxation, filling, diastolic distensibility and diastolic stiffness were found in the presence of normal or mildly reduced left ventricular systolic function (EF≥45%). Diastolic dysfunction was categorized into three levels as: *impaired relaxation* i.e. early diastolic dysfunction; *pseudonormal* i.e. a more severe condition indicating increasing left ventricular filling pressures or *restrictive filling pattern*. Diastolic dysfunction was then further dichotomized as being normal or not with impaired relaxation, pseudonormal or restrictive filling patterns categorized as diastolic dysfunction. Another senior cardiologist – not otherwise involved in the project and unaware of the findings in the first examination – reviewed the diagnostic criteria and the echocardiographic methods and re-examined a random sample (n=30) of the study sample using stored images. The results were consistent showing identical categorization.

Left ventricular mass was defined as: LVM = 0.8 × (1.04 [(left ventricular internal dimension + posterior wall thickness + septal thickness)^3 − (left ventricular internal dimension in diastole)^3]) + 0.6 g. LVM was then indexed for height$^{2.7}$(LVM-index), and LVH was defined as LVM-index ≥47.3 g/m $^{2.7}$ based on guidelines (26, 27).

Statistical analysis

Standard methods were used for descriptive statistics which were presented in terms of mean and standard deviation for all continuous variables and counts with percentage of categorical variables. All the statistical analysis were performed using program R 4.1.2. Possible confounding was addressed by stratification and multivariate analyses. Theoretical multiple logistic regressions were used to evaluate the association between diastolic dysfunction and alcohol consumption categories, with adjustments for different set of covariates. Model 1 includes only age and gender as traditional confounding factors. Model 2 adds smoking status and LTPA as lifestyle factors, which could be regarded as possible confounders or risk factors for outcome. Finally, Model 3 includes hypertension,

For peer review only - http://bmjopen.bmj.com/site/about/guidelines.xhtml

T2D, and BMI, which could be considered as mediators or strong determinants of diastolic dysfunction.

By presenting all three models the reader will have the possibility to judge the extent to which a change of the effect measure reflects removing a confounding effect and the extent to which the change of the effect measure reflects the degree of possible mediation.

These associations were expressed as odds ratios (OR) with 95% confidence intervals (CI). All tests were 2-sided and statistical significance were assumed at $p < 0.05$.

Ethical considerations

The investigation conforms to the principles outlined in the Declaration of Helsinki. The research ethics committee at the University of Gothenburg approved the study (DNR Ö199-01). The participants provided written informed consent to their participation in the health survey, and a further written informed consent was obtained before undergoing an ultrasound-Doppler examination of the heart.

Patient and Public Involvement

A meeting in the study center and the primary health care center was organized priory to the start of the study. A discussion with caregivers was organized to meet their concerns and ideas for the study but also to organize the care for individuals with pathological findings in the study. An open house event for the public was organized to inform and receive questions and suggestions. The results of the study are disseminated within the health care centers and health professionals.

Data availability statement

The data generated in the current study are available upon reasonable request, considering the sensitivity and confidentiality of the data. Due to the nature of the data, which contains personally identifiable information and sensitive information, it cannot be openly shared in a public repository.

For peer review only

Results

The mean age of the population was 55.4±13 years. In total, 79 of 500 men (16.5%) and 58 of 538 (12.9%) women were diagnosed with diastolic dysfunction (*p*=0.037). Totally 94% of the cases were categorized as *impaired relaxation* (74 men, 55 women) and 6% as *pseudonormal* (5 men, 3 women). There were no cases of severe *restrictive* filling patterns.

Alcohol consumption was common in both genders; men 84.5% (412/486) and women 68.8% (375/527), however, significant differences were observed between men and women with women being non-drinkers to a larger extent. In this observational study, non-drinkers were older, had higher blood pressure and had more often diabetes, however. Being allocated in one of groups based on alcohol consumption was not associated with diastolic dysfunction (p=0.500)*(Table1)*. Meanwhile, among subjects not using alcohol a higher rate of left ventricular hypertrophy (LVH) was observed. Characteristics of the study population as well as echocardiographic characteristics related to level of alcohol intake are presented in *Table 1*.

Table 1 Characteristics of participants related to level of alcohol consumption.

Variables	alcohol consumption<br>Non consumers<br>n=226 (22%)	Low<br>n=531 (53%)	Medium-High<br>n=254 (25%)	P*
Diastolic dysfunction[a]	35(15.5%)	66 (12.4%)	32 (12.6%)	0.500
Age (years)[b]	55.4(12.8)	49.5(11.5)	48 (10.7)	0.050
Women[a]	152 (67.3%)	289 (54.4%)	85 (33.5%)	<0.001
BMI (kg/m2)[b]	27.8(5.4)	26.8(4.2)	27 (3.9)	<0.001
SBP (mmHg)[b]	128.4(18.9)	123.6(17.1)	123.2 (15.6)	0.001
DBP (mmHg)[b]	71.1 (9.7)	71.1 (10.3)	72.1 (10.1)	0.300
HDL (mmol/L)[b]	1.2(0.3)	1.3(0.3)	1.3 (0.3)	0.100
TG (g/L)[b]	1.4(0.7)	1.3(0.8)	1.3 (0.7)	0.700
Level of physical activities				0.020
mobile	57(25.2%)	191(36.0%)	90(35.4%)	
medium	147(65%)	312(58.8%)	149(58.7%)	
sedentary	22(9.7%)	28(5.3%)	12(5.9%)	
Hypertension[a]	61 (27.0%)	93 (17.5%)	33 (13%)	<0.001
Diabetes[a]	29 (12.8%)	34 (6.4%)	10 (3.9%)	<0.001
Smoking habits[a]	39 (17.3%)	92 (17.3%)	54 (21.4%)	0.300

For peer review only - http://bmjopen.bmj.com/site/about/guidelines.xhtml

E/A ratio	1.2(0.4)	1.4(0.4)	1.4(0.4)	<0.001
IVRT	98.4(21.9)	91.6(20.1)	93.2(21.2)	<0.001
EJECTION FRACTION	72.2(9.6)	74.4(8.4)	74.4(7.8)	0.005
Septal wall (cm)	1(0.2)	0.9(0.2)	0.9(0.2)	0.008
Posterior wall (cm)	1(0.3)	0.9(0.2)	1(0.5)	0.010
LAD (cm)	3.3(0.5)	3.2(0.5)	3.3(0.5)	0.040
LV-mass (Median and 5th, 95th percentiles)	37.8 (23.4, 62)	34.5 (23.8, 55.6)	35.1 (23.9, 57.5)	0.050*
LVH	52 (23.0%)	70(13.2%)	32(12.6%)	0.001#

[a] data is presented as counts (percent).[b] data are presented with mean (standard deviation)

*P values are based ANOVA if the variables are continuous or based on chi-square test if the variables are binary. Medium-high is defined as consumption >56 g/week and low those with alcohol consumption ≤56g/week. BMI, Body mass index, SBP, Systolic blood pressure, DBP Diastolic blood pressure, HDL, High density lipoprotein- cholesterol, TG, Triglycerides, IVRT Isovolumic relaxation time, LAD-left atrial diameter

In a multivariable theoretical model, adjusted for possible confounders and mediators in the association between alcohol consumption and diastolic dysfunction, we found that alcohol consumption was significantly associated with increased risk of diastolic dysfunction. Interestingly, even low alcohol consumption was strongly associated with increased risk for diastolic dysfunction (OR=2.2 95% CI 1.3-3.9). The estimates were even higher for participants with moderate to high alcohol consumption (OR=3.1 95% CI 1.6-6.2) with a strong significant trend (p=0.001). Similar results were observed when adjustments for systolic blood pressure were included in the final models (Low consumption: OR= 2.3 95% CI 1.3-4.1; moderate to high alcohol consumption OR= 3.1 95% CI 1.6-6.2). In another model we tested also to adjust in the final model for HDL-cholesterol on the top of adjustments for age, gender, hypertension, type 2 diabetes, BMI, smoking habits and leisure time physical activities and the association was still strong and significant (Low consumption: OR= 2.4 95% CI 1.4-4.4; moderate to high alcohol consumption OR= 3.4 95% CI 1.7-6.9). Exploring in the same models the association between alcohol consumption and left atrial diameter no significant differences were observed (data not shown). There were no significant differences in these associations were observed between men and women (p for interaction >0.25). High age, male sex,

For peer review only - http://bmjopen.bmj.com/site/about/guidelines.xhtml

hypertension, high BMI, and a low level of leisure time physical activity were all also independently associated with diastolic dysfunction (*Table 2)*.

Table 2: Association between risk factors and diastolic dysfunction

	OR	95% confidence interval
**Model 1**		
Age	1.16	1.13-1.19
Men vs Women	1.34	0.87-2.07
Alcohol consumption		
None	1	Ref
Low vs none	1.82	1.08-3.12
High vs none	2.39	1.26-4.58
**Model 2**		
Age	1.16	(1.13, 1.19)
Men vs Women	1.46	(0.94, 2.27)
Current smoking	1.39	(0.78, 2.42)
physical activity		
active	1	Ref
medium vs active	1.6	(0.97, 2.69)
sedentary vs active	4.4	(1.93, 9.96)
alcohol consumption	1	Ref
low vs non	2.03	(1.19, 3.5)
high vs non	2.68	(1.4, 5.19)
**Model 3**		
Age	1.14	(1.11-1.18)
Men vs Women	1.63	(1.02-2.63)
BMI	1.07	(1.01-1.11)
Hypertension	2.68	(1.65-4.33)
Type 2 Diabetes	1.88	(1.00-3.53)
Current smoking	1.76	(0.96-3.15)
Leisure time physical activity		
medium vs active	1.28	(0.75-2.23)
sedentary vs active	2.82	(1.15-6.84)
Alcohol consumption		
None	1	Ref
low vs none	2.25	(1.29-4.02)
high vs none	3.06	(1.55-6.15)

Multivariable logistic regression analyses are used to investigate the association between explanatory variables and diastolic dysfunction. OR Odds ratio, BMI body mass index. In model 1 adjustments for age and gender, in model 2 adjustments for age, gender, current smoking, leisure

For peer review only

time physical activities, in model 3 adjustments for age, gender, BMI, hypertension, type 2 diabetes, current smoking, leisure time physical activities are computed. Women are the reference group in the analyses men vs women and physically active group is the reference in the analyses of leisure time physical activity

Similar analyses were computed using LVH as outcome, but no significant association was found with alcohol consumption (*Table3*).

Table 3: Association between risk factors and left ventricular hypertrophy

	OR	confidence interval
Age	1.08	(1.06-1.1)
Men vs Women	1.16	(0.75-1.78)
BMI	1.16	(1.11-1.22)
Hypertension	3.20	(2.05-5.01)
Type 2 Diabetes	1.30	(0.70-2.38)
Current smoking	1.53	(0.89-2.57)
Leisure time physical activity		
medium vs mobile	0.96	(0.60-1.56)
sedentary vs mobile	1.18	(0.50-2.69)
Alcohol consumption		
Non drinkers	1	Ref
low vs non	1.08	(0.59-1.98)
high vs non	1.53	(0.89-2.57)

Multivariable logistic regression analyses are used to investigate the association between explanatory variables and left ventricular hypertrophy. OR Odds ratio, BMI body mass index

For peer review only

For peer review only - http://bmjopen.bmj.com/site/about/guidelines.xhtml

Discussion

In this cross-sectional observational study, we found a strong and independent association between self-reported alcohol consumption and diastolic dysfunction. Interestingly, our data show an increased risk of having diastolic dysfunction already in subjects with low-level of alcohol consumption. Moreover, in gender specific analyses no gender differences were observed suggesting a similar effect of alcohol in both men and women. Despite these findings, no significant association between alcohol intake and LVH were observed. Like previous observations, age, gender, hypertension, leisure time physically activity levels, and BMI were all strongly and independently associated with diastolic dysfunction.

These findings are not in line with most of the observations on the role of alcohol in heart failure. In fact, population-based studies have shown that *moderate* levels of alcohol consumption (i.e. <1 drinks per day (d/d) in females and <2 d/d in males) have a protective effect on ischemic heart disease and decreases the incidence of CHF (1, 28) (3). The beneficial effect of a low to moderate amount of alcoholic intake are not reported by all epidemiological studies (29, 30). A 'healthy cohort effect' is reported as one plausible explanation to the favourable effects of moderate consumption (29, 30) where the confounding effects of for example physical activity, mental health, psychosocial, socioeconomic and social network related factors seldom are accounted for (30). Moreover, in a recent observation in HUNT study (31) no protective effect of low-alcohol consumption on left ventricular function was observed, however, a positive linear association was observed between alcohol consumption and left ventricular mass and end diastolic diameter. The findings of HUNT-study are in line with our findings however the significance in the association was lost in their multivariable analyses. Interestingly, before the adjustments in our cohort higher prevalence of diastolic dysfunction was observed in nondrinkers. These differences were not significant. The same group had also higher age, lower level of physical activities, higher systolic blood pressure and higher prevalence of hypertension and diabetes. It was first after the adjustments for these factors that a positive linear association between alcohol consumption and diastolic dysfunction was observed. These findings suggest no protective effect of alcohol at low

For peer review only - http://bmjopen.bmj.com/site/about/guidelines.xhtml

levels. Also, alcohol seems not to be related with better diastolic dysfunction, but healthy and younger subjects are more prone to drink alcohol and they have of course better diastolic function but not of alcohol consumption.

Several alcohol metabolites are identified as particularly toxic to the myocardium: ethanol, acetaldehyde (the metabolic product of alcohol) and fatty acid ethyl esters (32, 33). Alcohol also impairs the transport of calcium ions from the sarcoplasma to the sarcoplasmatic reticulum (34, 35) resulting in diastolic dysfunction characterized by delayed myocardial relaxation (36) and impaired left ventricular (LV) diastolic filling. Moderate versus high doses of alcohol affect myocardial function differently through oxidative stress, haemostatic factors, levels of C-reactive protein, interleukins, and TNF alpha (29).

There have been observations that suggest that patterns of drinking and food intake might explain at least partially the damages of alcohol in myocardium(5, 37, 38). In fact, the Mediterranean countries with a low prevalence of CVD, alcohol is commonly consumed with meals as compared to Russia with a high prevalence of CVD where binge drinking is much more frequent (30). However, some studies have shown there is evidence that alcoholic cardiomyopathy can be developed even in absence of malnutrition(39) while these deleterious effects are exaggerated of malnutrition(40, 41).

Interestingly, our results did not show any significant association with LVH. One reason of the lack of association might be the relatively low number of individuals with high consumption in this cohort. Measurable structural remodelling might need higher level of alcohol consumption. In fact, a large population study in South Korea with significantly larger number of participants compared with our study, could find a strong association between alcohol consumption and LVH(42).

Strengths and limitations

Echocardiography was used in this large population-based cohort using standardized age-dependent criteria to identify diastolic disfunction based on recommended by international expert groups. The re-evaluation of a sub-sample by a senior cardiologist confirmed the first assessment and the consistency in assessment of prevalence of diastolic dysfunction and in identified risk factors between our findings and other

For peer review only - http://bmjopen.bmj.com/site/about/guidelines.xhtml

comprehensive surveys using Doppler techniques, lend further support to the validity of our data.

The characteristics of alcohol consumers and prevalence of alcohol consumption was in accordance with other comparable community-based surveys (43, 44). This, however, might be a source of underestimation of the alcohol consumption. In fact, in several studies there is an underestimation of self-reported alcohol consumption(45) and there is need of more objective and reliable measures of alcohol consumption. However, the underestimation should be more frequent and mostly in the group with higher consumption. This would not have an impact effect on our results in the association found between low alcohol consumption and diastolic dysfunction. Moreover, the findings are based on a rural population in Sweden and differences in these association might appear when other population are considered however the consistency of the results with previous findings indicate that these findings might apply in other populations. Finally, this was a cross sectional observation and no conclusions in causality can be drawn and the residual confounding is not possible to rule out.

Conclusion

Our study revealed a significant association between alcohol consumption and diastolic dysfunction starting already at low levels that was supported by a dose-dependent pattern. More prospective surveys with adequate measurement of alcohol consumption and possible confounders are needed for a better understanding of the underlying causality and mechanisms. These results are important on the public health policies and information on alcohol use.

Competing interests

The authors declare that they have no competing interests.

Acknowledgements and funding

We are indebted to all participants in the Vara Cohort. Without them this study would not been possible. The Knut and Alice Wallenberg foundation is recognized for generous support. This work was supported by: The Swedish Science Council; Sahlgrenska Academy, University of Gothenburg; Skaraborg Institute; The Health & Medical Care

For peer review only - http://bmjopen.bmj.com/site/about/guidelines.xhtml

Committee of the Regional Executive Board of the Region Västragötalands region VGFOUREG-86294, VGFOUREG-969459, ALFGBG-966255, ALFGBG-971147; Skaraborg Primary Care; Skane Region; Lund University; University Hospital of Malmoe donation Foundation; Påhlsson Foundation; Crafoord Foundation; Lundberg Foundation, the Swedish Heart and Lung foundation and the Wallenberg Center for Molecular Medicine, Lund University; and Lund University. The funding source was not involved in the work with the article.

Author contribution: All authors read and approved the final manuscript. Research idea and study design: LB, BD, UL, MH; Data acquisition: UL, LR, BD, MH; Data analysis and interpretation: LB, UL, BD, MM, MH; Statistical analyses: LB, BD, YL, UL. Each author contributed important intellectual content during manuscript drafting or revision, accepts personal accountability for the author's own contributions, and agrees to ensure that questions pertaining to the accuracy or integrity of any portion of the work are appropriately investigated and resolved.

For peer review only

For peer review only - http://bmjopen.bmj.com/site/about/guidelines.xhtml

References

1.Djousse, L. and Gaziano, J.M. (2007). Alcohol consumption and risk of heart failure in the Physicians' Health Study I. Circulation 115: 34-39.
2.World Health Organization. Management of Substance Abuse Team. Global status report on alcohol and health, pvolumes, World Health Organization, Geneva, Switzerland.
3.Zhong, L., Chen, W., Wang, T., Zeng, Q., Lai, L., Lai, J., Lin, J. and Tang, S. (2022). Alcohol and Health Outcomes: An Umbrella Review of Meta-Analyses Base on Prospective Cohort Studies. Front Public Health 10: 859947.
4.Larsson, S.C., Wallin, A. and Wolk, A. (2018). Alcohol consumption and risk of heart failure: Meta-analysis of 13 prospective studies. Clin Nutr 37: 1247-1251.
5.Murray, R.P., Connett, J.E., Tyas, S.L., Bond, R., Ekuma, O., Silversides, C.K. and Barnes, G.E. (2002). Alcohol volume, drinking pattern, and cardiovascular disease morbidity and mortality: is there a U-shaped function? Am J Epidemiol 155: 242-248.
6.Lazarevic, A.M., Nakatani, S., Neskovic, A.N., Marinkovic, J., Yasumura, Y., Stojicic, D., Miyatake, K., Bojic, M. and Popovic, A.D. (2000). Early changes in left ventricular function in chronic asymptomatic alcoholics: relation to the duration of heavy drinking. J Am Coll Cardiol 35: 1599-1606.
7.(1998). How to diagnose diastolic heart failure. European Study Group on Diastolic Heart Failure. Eur Heart J 19: 990-1003.
8.Fischer, M., Baessler, A., Hense, H.W., Hengstenberg, C., Muscholl, M., Holmer, S., Doring, A., Broeckel, U., Riegger, G. and Schunkert, H. (2003). Prevalence of left ventricular diastolic dysfunction in the community. Results from a Doppler echocardiographic-based survey of a population sample. Eur Heart J 24: 320-328.
9.Redfield, M.M., Jacobsen, S.J., Burnett, J.C., Jr., Mahoney, D.W., Bailey, K.R. and Rodeheffer, R.J. (2003). Burden of systolic and diastolic ventricular dysfunction in the community: appreciating the scope of the heart failure epidemic. JAMA 289: 194-202.
10.Vogel, M.W., Slusser, J.P., Hodge, D.O. and Chen, H.H. (2012). The natural history of preclinical diastolic dysfunction: a population-based study. Circ Heart Fail 5: 144-151.
11.Catena, C., Colussi, G., Verheyen, N.D., Novello, M., Fagotto, V., Soardo, G. and Sechi, L.A. (2016). Moderate Alcohol Consumption Is Associated With Left Ventricular Diastolic Dysfunction in Nonalcoholic Hypertensive Patients. Hypertension 68: 1208-1216.
12.Liu, F., Liu, Y., Sun, X., Yin, Z., Li, H., Deng, K., Zhao, Y., Wang, B., Ren, Y., Liu, X., Zhang, D., Chen, X., Cheng, C., Liu, L., Liu, D., Chen, G., Hong, S., Wang, C., Zhang, M. and Hu, D. (2020). Race- and sex-specific association between alcohol consumption and hypertension in 22 cohort studies: A systematic review and meta-analysis. Nutr Metab Cardiovasc Dis 30: 1249-1259.
13.Chakraborty, S. (2014). Analysis of NHANES 1999-2002 data reveals noteworthy association of alcohol consumption with obesity. Ann Gastroenterol 27: 250-257.
14.Larsson, C.A., Kroll, L., Bennet, L., Gullberg, B., Rastam, L. and Lindblad, U. (2012). Leisure time and occupational physical activity in relation to obesity and insulin resistance: a population-based study from the Skaraborg Project in Sweden. Metabolism 61: 590-598.

For peer review only - http://bmjopen.bmj.com/site/about/guidelines.xhtml

15.Ingelsson, E., Bennet, L., Ridderstrale, M., Soderstrom, M., Rastam, L. and Lindblad, U. (2008). The PPARGC1A Gly482Ser polymorphism is associated with left ventricular diastolic dysfunction in men. BMC Cardiovasc Disord 8: 37.
16.(1999). 1999 World Health Organization-International Society of Hypertension Guidelines for the Management of Hypertension. Guidelines Subcommittee. J Hypertens 17: 151-183.
17.Alberti, K.G. and Zimmet, P.Z. (1998). Definition, diagnosis and classification of diabetes mellitus and its complications. Part 1: diagnosis and classification of diabetes mellitus provisional report of a WHO consultation. Diabet Med 15: 539-553.
18.Goransson, M. and Hanson, B.S. (1994). How much can data on days with heavy drinking decrease the underestimation of true alcohol consumption? J Stud Alcohol 55: 695-700.
19.Bantle, J.P., Wylie-Rosett, J., Albright, A.L., Apovian, C.M., Clark, N.G., Franz, M.J., Hoogwerf, B.J., Lichtenstein, A.H., Mayer-Davis, E., Mooradian, A.D. and Wheeler, M.L. (2006). Nutrition recommendations and interventions for diabetes--2006: a position statement of the American Diabetes Association. Diabetes Care 29: 2140-2157.
20.Nyholm, M., Gullberg, B., Haglund, B., Rastam, L. and Lindblad, U. (2008). Higher education and more physical activity limit the development of obesity in a Swedish rural population. The Skaraborg Project. Int J Obes (Lond) 32: 533-540.
21.Lang, R.M., Bierig, M., Devereux, R.B., Flachskampf, F.A., Foster, E., Pellikka, P.A., Picard, M.H., Roman, M.J., Seward, J., Shanewise, J.S., Solomon, S.D., Spencer, K.T., Sutton, M.S. and Stewart, W.J. (2005). Recommendations for chamber quantification: a report from the American Society of Echocardiography's Guidelines and Standards Committee and the Chamber Quantification Writing Group, developed in conjunction with the European Association of Echocardiography, a branch of the European Society of Cardiology. J Am Soc Echocardiogr 18: 1440-1463.
22.Jarnert, C., Mejhert, M., Ring, M., Persson, H. and Edner, M. (2000). Doppler tissue imaging in congestive heart failure patients due to diastolic or systolic dysfunction: a comparison with Doppler echocardiography and the atrio-ventricular plane displacement technique. Eur J Heart Fail 2: 151-160.
23.Gudmundsson, P., Rydberg, E., Winter, R. and Willenheimer, R. (2005). Visually estimated left ventricular ejection fraction by echocardiography is closely correlated with formal quantitative methods. Int J Cardiol 101: 209-212.
24.Oh, J.K., Appleton, C.P., Hatle, L.K., Nishimura, R.A., Seward, J.B. and Tajik, A.J. (1997). The noninvasive assessment of left ventricular diastolic function with two-dimensional and Doppler echocardiography. J Am Soc Echocardiogr 10: 246-270.
25.Swedberg, K., Cleland, J., Dargie, H., Drexler, H., Follath, F., Komajda, M., Tavazzi, L., Smiseth, O.A., Gavazzi, A., Haverich, A., Hoes, A., Jaarsma, T., Korewicki, J., Levy, S., Linde, C., Lopez-Sendon, J.L., Nieminen, M.S., Pierard, L. and Remme, W.J. (2005). Guidelines for the diagnosis and treatment of chronic heart failure: executive summary (update 2005): The Task Force for the Diagnosis and Treatment of Chronic Heart Failure of the European Society of Cardiology. Eur Heart J 26: 1115-1140.
26.de Simone, G., Devereux, R.B., Chinali, M., Roman, M.J., Lee, E.T., Resnick, H.E. and Howard, B.V. (2009). Metabolic syndrome and left ventricular hypertrophy in the prediction of cardiovascular events: the Strong Heart Study. Nutr Metab Cardiovasc Dis 19: 98-104.

For peer review only

For peer review only - http://bmjopen.bmj.com/site/about/guidelines.xhtml

27.Krauser, D.G. and Devereux, R.B. (2006). Ventricular hypertrophy and hypertension: prognostic elements and implications for management. Herz 31: 305-316.
28.Walsh, C.R., Larson, M.G., Evans, J.C., Djousse, L., Ellison, R.C., Vasan, R.S. and Levy, D. (2002). Alcohol consumption and risk for congestive heart failure in the Framingham Heart Study. Ann Intern Med 136: 181-191.
29.Bau, P.F., Bau, C.H., Rosito, G.A., Manfroi, W.C. and Fuchs, F.D. (2007). Alcohol consumption, cardiovascular health, and endothelial function markers. Alcohol 41: 479-488.
30.Fuchs, F.D. and Chambless, L.E. (2007). Is the cardioprotective effect of alcohol real? Alcohol 41: 399-402.
31.Gemes, K., Janszky, I., Strand, L.B., Laszlo, K.D., Ahnve, S., Vatten, L.J., Dalen, H. and Mukamal, K.J. (2018). Light-moderate alcohol consumption and left ventricular function among healthy, middle-aged adults: the HUNT study. BMJ Open 8: e020777.
32.Beckemeier, M.E. and Bora, P.S. (1998). Fatty acid ethyl esters: potentially toxic products of myocardial ethanol metabolism. J Mol Cell Cardiol 30: 2487-2494.
33.Ren, J. and Wold, L.E. (2008). Mechanisms of alcoholic heart disease. Ther Adv Cardiovasc Dis 2: 497-506.
34.Cofan, M., Nicolas, J.M., Fernandez-Sola, J., Robert, J., Tobias, E., Sacanella, E., Estruch, R. and Urbano-Marquez, A. (2000). Acute ethanol treatment decreases intracellular calcium-ion transients in mouse single skeletal muscle fibres in vitro. Alcohol Alcohol 35: 134-138.
35.Noren, G.R., Staley, N.A., Einzig, S., Mikell, F.L. and Asinger, R.W. (1983). Alcohol-induced congestive cardiomyopathy: an animal model. Cardiovasc Res 17: 81-87.
36.Kupari, M., Koskinen, P., Suokas, A. and Ventila, M. (1990). Left ventricular filling impairment in asymptomatic chronic alcoholics. Am J Cardiol 66: 1473-1477.
37.Urquiaga, I., Guasch, V., Marshall, G., San Martin, A., Castillo, O., Rozowski, J. and Leighton, F. (2004). Effect of Mediterranean and Occidental diets, and red wine, on plasma fatty acids in humans. An intervention study. Biol Res 37: 253-261.
38.Britton, A. and McKee, M. (2000). The relation between alcohol and cardiovascular disease in Eastern Europe: explaining the paradox. J Epidemiol Community Health 54: 328-332.
39.Urbano-Marquez, A., Estruch, R., Navarro-Lopez, F., Grau, J.M., Mont, L. and Rubin, E. (1989). The effects of alcoholism on skeletal and cardiac muscle. N Engl J Med 320: 409-415.
40.Fernandez-Sola, J. (2015). Cardiovascular risks and benefits of moderate and heavy alcohol consumption. Nat Rev Cardiol 12: 576-587.
41.Piano, M.R. and Phillips, S.A. (2014). Alcoholic cardiomyopathy: pathophysiologic insights. Cardiovasc Toxicol 14: 291-308.
42.Park, S.K., Moon, K., Ryoo, J.H., Oh, C.M., Choi, J.M., Kang, J.G., Chung, J.Y. and Young Jung, J. (2018). The association between alcohol consumption and left ventricular diastolic function and geometry change in general Korean population. Eur Heart J Cardiovasc Imaging 19: 271-278.
43.Bostrom, G. and Nykvist, K. (2004). Lifestyle and health (in Swedish), The National Institute of Public Health in Sweden. Available at *http://www.fhi.se/upload/ar2005/rapporter/r200448levnadsvanorhalsa0504.pdf*

For peer review only

44. Wilsgaard, T. and Jacobsen, B.K. (2007). Lifestyle factors and incident metabolic syndrome. The Tromso Study 1979-2001. Diabetes Res Clin Pract 78: 217-224.
45. Stockwell, T., Zhao, J., Sherk, A., Rehm, J., Shield, K. and Naimi, T. (2018). Underestimation of alcohol consumption in cohort studies and implications for alcohol's contribution to the global burden of disease. Addiction 113: 2245-2249.

For peer review only

For peer review only - http://bmjopen.bmj.com/site/about/guidelines.xhtml

For peer review only

For peer review only - http://bmjopen.bmj.com/site/about/guidelines.xhtml

Figure 1. Study population in the Vara Cohort 2002-2003

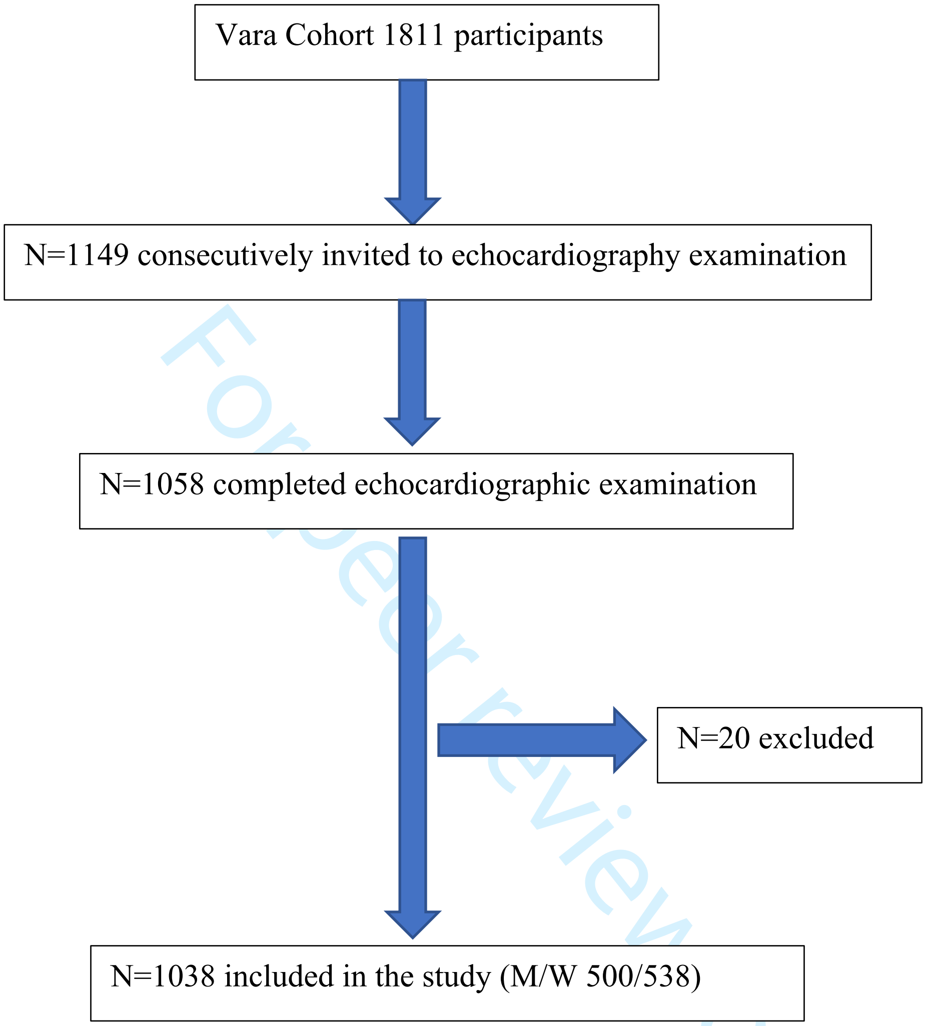

*Cases excluded due to at least one of the following reasons: arrhythmia/atrial fibrillation n=8, physical characteristics hindering UCG n=6, self-reported history of heart failure n=6, EF<45% n=2 and/or aortic insufficiency n=2.

For peer review only

BMJ Open

Association between self-reported alcohol consumption and diastolic dysfunction, a cross-sectional study

Journal:	*BMJ Open*
Manuscript ID	bmjopen-2022-069937.R2
Article Type:	Original research
Date Submitted by the Author:	08-Sep-2023
Complete List of Authors:	Daka, Bledar; University of Gothenburg Sahlgrenska Academy, Medicine<br>Bennet, Louise; Lund University, Family Medicine, Deaprtment of Clinical Sciences<br>Råstam, Lennart ; Lund University, Department of Clinical Sciences, Malmö, Sweden<br>Hellgren, Margareta; University of Gothenburg<br>Li, Ying; Gothenburg University Library<br>Magnusson, Martin; Lund University<br>Lindblad, Ulf; Lund University, Department of Clinical Sciences in Malmö
**Primary Subject Heading**:	Cardiovascular medicine
Secondary Subject Heading:	General practice / Family practice, Epidemiology
Keywords:	Echocardiography < CARDIOLOGY, PREVENTIVE MEDICINE, Heart failure < CARDIOLOGY

SCHOLARONE™
Manuscripts

I, the Submitting Author has the right to grant and does grant on behalf of all authors of the Work (as defined in the below author licence), an exclusive licence and/or a non-exclusive licence for contributions from authors who are: i) UK Crown employees; ii) where BMJ has agreed a CC-BY licence shall apply, and/or iii) in accordance with the terms applicable for US Federal Government officers or employees acting as part of their official duties; on a worldwide, perpetual, irrevocable, royalty-free basis to BMJ Publishing Group Ltd ("BMJ") its licensees and where the relevant Journal is co-owned by BMJ to the co-owners of the Journal, to publish the Work in this journal and any other BMJ products and to exploit all rights, as set out in our licence.

The Submitting Author accepts and understands that any supply made under these terms is made by BMJ to the Submitting Author unless you are acting as an employee on behalf of your employer or a postgraduate student of an affiliated institution which is paying any applicable article publishing charge ("APC") for Open Access articles. Where the Submitting Author wishes to make the Work available on an Open Access basis (and intends to pay the relevant APC), the terms of reuse of such Open Access shall be governed by a Creative Commons licence – details of these licences and which Creative Commons licence will apply to this Work are set out in our licence referred to above.

Other than as permitted in any relevant BMJ Author's Self Archiving Policies, I confirm this Work has not been accepted for publication elsewhere, is not being considered for publication elsewhere and does not duplicate material already published. I confirm all authors consent to publication of this Work and authorise the granting of this licence.

For peer review only

Association between self-reported alcohol consumption and diastolic dysfunction, a cross-sectional study

Bledar Daka MD MD PhD[1], Louise Bennet MD, PhD[2, 3], Lennart Råstam MD, PhD[2], Margareta I Hellgren MD PhD [1, 5], Ying Li PhD[1], Martin Magnusson MD PhD[2,6, 7, 8], Ulf Lindblad MD, PhD[1, 5]

[1] Family medicine, School of Public Health and Community Medicine, The Sahlgrenska Academy, the University of Gothenburg, Sweden
[2] Lund University, Department of Clinical Sciences, Malmö, Sweden
[3] Clinical Trial Unit, Skåne University Hospital, Lund, Sweden
[4] Department of Cardiology, Skaraborg Hospital, Skövde, Sweden
[5] Skaraborg Institute, Skövde, Sweden
[6] Department of Cardiology, Skåne University Hospital, Malmö, Sweden
[7] Hypertension in Africa Research Team (HART); North-West University, Potchefstroom, South Africa
[8] Wallenberg Center for Molecular Medicine, Lund University, Lund, Sweden

Correspondence to: Bledar Daka Associated Professor, School of Public Health and Community Medicine/Primary Health Care, Hälsovetarbacken 7, Box 454, SE 405 30 Göteborg, Sweden
Phone: +46 31 786 68 31
e-mail: bledar.daka@allmed.gu.se

For peer review only

Abstract

Background and objectives: While alcohol consumption is associated with common risk factors for diastolic dysfunction the independent impact of low levels of alcohol consumption on this condition in a community setting is still unclear.

Thus, the aim of this study was to explore this association in a representative population sample employing optimal echocardiographic techniques.

Design: Cross-sectional observational study in community-based population

Settings, participants, and methods: Participants between 30 to 75 years of age were consecutively invited to a physical exam, interview, conventional echocardiography, including Tissue Velocity Imaging. Diastolic dysfunction was defined according to the European Society of Cardiology criteria, excluding subjects with ejection fraction <45%, self-reported history of heart failure, or atrial fibrillation on ECG. Self-reported alcohol intake using a validated questionnaire was categorized as *no intake, low and medium-high* intake.

Results: In total, 500 men and 538 women (Mean age 55.4±13) were successfully examined. Diastolic dysfunction was identified in 16% (79/500) of the men and 13% (58/538) of the women. The multivariable adjusted model revealed a strong and independent association between alcohol intake and diastolic dysfunction. In fact, using no alcohol intake as reference, diastolic dysfunction was independently associated with alcohol consumption in a dose-dependent fashion; *low consumption,* OR 2.3 (95% CI 1.3 to 4.0) and *medium-high consumption OR* 3.1 (95% CI 1.6 to 6.2), respectively.

Conclusion: There was a significant association between alcohol consumption and diastolic dysfunction starting already at low levels that was supported by a dose-dependent pattern. These results need confirmatory studies and are important in public health policies.

Keywords: *diastolic dysfunction, echocardiography, heart failure, alcohol consumption*, public health

For peer review only - http://bmjopen.bmj.com/site/about/guidelines.xhtml

Strengths and limitations of the study

1. Echocardiography was performed in a large population-based cohort using standardized methods. The re-evaluation of a sub-sample by a senior cardiologist confirmed the first assessment and the consistency.
2. Detailed information on other risk factors including oral glucose tolerance test to define diabetes mellitus was available.
3. Validated questionnaires on alcohol consumption was used, however, still self-reported.
4. Finally, this was a cross sectional observational study and no conclusions in causality can be drawn. However, randomized controlled trials in this field are and will remain difficult to perform.

For peer review only

Introduction

Based on World Health Organisation from 2018 about 57% of adult population globally are alcohol consumers and 3 million deaths are attributed to alcohol consumption in 2016 (1, 2). A recently published meta-analysis could identify better health outcomes in people that had reported low to moderate alcohol consumption (3). On the other hand, several studies have shown that moderate levels of alcohol (i.e. alcohol equalling <1 drink per day (d/d) of alcohol in females and <2 d/d in males) have a protective effect on ischemic heart disease and chronic heart failure (CHF)(1, 4) while heavy episodic drinking negates from these effects (5). Interestingly, chronic excessive alcohol consumption is also reported to be associated with diastolic dysfunction (6), which is characterized by disturbed relaxation and increased preload. Diastolic dysfunction represents one out of three criteria for diastolic heart failure (7) and occurs often without impaired physical performance or other symptoms. The condition is common with prevalence in community-based studies varying from a few to 30 per cent (8, 9) and is associated with increase in risk to develop heart failure (10)

In patients with hypertension, moderate alcohol consumption have been associated with diastolic dysfunction (11). Although alcohol consumption is associated with common risk factors for diastolic dysfunction as hypertension(12) and obesity(13) the independent impact of low to levels of alcohol consumption on this condition in a community setting is still unclear. While there is consensus on deleterious effects of heavy drinking, the role of low alcohol consumption on CVD and CHF risk and diastolic dysfunction is unknown. Also, the mechanisms of a protective effect in low doses and deleterious effect in higher doses haven't been explained yet.

Thus, the objective of this study was to explore the associations between alcohol intake and asymptomatic diastolic dysfunction with preserved systolic function in a population-based cohort.

For peer review only - http://bmjopen.bmj.com/site/about/guidelines.xhtml

Materials and Methods

Subjects

This study includes data from a surveillance of the population in Vara 2002–2003, a small community with 16 000 residents in a rural area of south-western Sweden(14). The sampling, inclusion of participants and echocardiography-examination was conducted as previously described (15). Briefly, men (500) and women (538) 30 to 75 years were selected from the census register and invited to participate. Individuals below age 50 years were over-sampled three times as compared to those who were older. Participation was based on conducting an echocardiography and physical examination, filling in questionnaires and returning blood samples. Subjects with ejection fraction <45%, self-reported history of heart failure, arrhythmia, aortic insufficiency, or physical characteristics hindering echo were excluded from the analyses (N=20). The study profile is presented in figure 1.

Demographic and Lifestyle Data

Standardized physical examinations were performed and blood samples drawn including oral glucose tolerance tests (15). Blood pressure was measured in lying position after 5 minutes rest. Two measurements were done with 1 minute apart and the mean value was used. Diagnosis of hypertension was defined in accordance with international guidelines based on medical history of hypertension or three consecutive blood pressure measurements equal or higher than 140/90 mmHg (16). Diabetes was defined based on World Health Organisation recommendations (17). Information on previous cardiovascular disease (CVD) was collected through questionnaires. Information on smoking habits was also collected through questionnaires and participants were categorized as never smokers, former smokers, and current smokers.

Alcohol intake*:* Participants were asked to estimate the number of days they had been drinking alcohol the last month and the average amount of alcohol they had consumed during those days. Based on validated questionaries(18) for self-reported alcohol intake an estimation on gram per week of alcohol consumption was calculated for each participant. Based on quartiles of this estimated weekly alcohol consumption, the

For peer review only - http://bmjopen.bmj.com/site/about/guidelines.xhtml

participants were divided into 3 groups. Non-drinkers were participants that reported no alcohol consumption the last month. Highest quartile (>56 g/week) was defined as medium-high consumers and those with alcohol consumption ≤56g/week were defined as low consumers. One drink of alcohol was estimated equivalent to 15 grams of alcohol (19).

Level of leisure time physical activity (LTPA) was self-reported in four possible levels: *Sedentary LTPA* i.e. mostly physically inactive, *less strenuous LTPA* (walks, cycling, gardening etc.) <4 hours a week (h/w); *Medium LTPA* i.e. less strenuous LTPA ≥4h/w or *High LTPA* i.e. strenuous LTPA (jogging, swimming, tennis etc.) ≥2 h/w (20). Due to reduced number of individuals in the strenuous category we grouped group 3 and 4 together and defined it as reference.

Echocardiographic methods

All participants were examined by the same senior cardiologist, using echocardiography (General Electrics VingMed S 5 System, operating with a 3.5 MHz-probe) (15). The same cardiologist performed all the measurements and had no access on information on alcohol drinking habits. Data was stored in the EchoPac System for playback analysis and measurement. Measurements for left ventricular calculations were taken from the Guidelines of the American Society of Echocardiography (21). A two-dimensional echocardiogram was formed from the left parasternal and apical windows for measurements. The EF was calculated from apical four and two chamber views with longitudinal systolic shortening mean index/ atrio-ventricular plane displacement technique in addition to a semi-quantitative visual estimate method (22, 23). The ventricular diastolic function was based on several parameters and their respective normal ranges were defined for different age categories (24). The E-wave-peak (early filling) to A-wave-peak (atrial filling), and the ratio were then determined. The isovolumetric relaxation time (IVRT), from the closure of the aortic valve to the opening of the mitral valve, was also measured. To indirectly measure distensibility in the left ventricle, the deceleration time (DT), the peak E to baseline-slope, was measured. Tissue Velocity Imaging (TVI) was used in both the septum and in the more stable lateral wall to

For peer review only

detect pseudo-normalization. The patient was also told to perform the Valsalva manoeuvre during registration.

Diastolic dysfunction was defined according to the recommendations of the European Society of Cardiology(24, 25) when evidence of abnormal left ventricular diastolic relaxation, filling, diastolic distensibility and diastolic stiffness were found in the presence of normal or mildly reduced left ventricular systolic function (EF≥45%). Diastolic dysfunction was categorized into three levels as: *impaired relaxation* i.e. early diastolic dysfunction; *pseudonormal* i.e. a more severe condition indicating increasing left ventricular filling pressures or *restrictive filling pattern*. Diastolic dysfunction was then further dichotomized as being normal or not with impaired relaxation, pseudonormal or restrictive filling patterns categorized as diastolic dysfunction. Another senior cardiologist – not otherwise involved in the project and unaware of the findings in the first examination – reviewed the diagnostic criteria and the echocardiographic methods and re-examined a random sample (n=30) of the study sample using stored images. The results were consistent showing identical categorization.

Left ventricular mass was defined as: LVM = 0.8 × (1.04 [(left ventricular internal dimension + posterior wall thickness + septal thickness)^3 − (left ventricular internal dimension in diastole)^3]) + 0.6 g. LVM was then indexed for height$^{2.7}$(LVM-index), and LVH was defined as LVM-index ≥47.3 g/m $^{2.7}$ based on guidelines (26, 27).

Statistical analysis

Standard methods were used for descriptive statistics which were presented in terms of mean and standard deviation for all continuous variables and counts with percentage of categorical variables. All the statistical analysis were performed using program R 4.1.2. Possible confounding was addressed by stratification and multivariate analyses. Theoretical multiple logistic regressions were used to evaluate the association between diastolic dysfunction and alcohol consumption categories, with adjustments for different set of covariates. In the theoretical models we included traditional confounding factors as age and gender. Current smoking status and LTPA are lifestyle factor with strong covariation with alcohol consumption and that can influence the risk for diastolic dysfunction. Traditional determinants of diastolic dysfunction as HT, T2D and obesity

For peer review only - http://bmjopen.bmj.com/site/about/guidelines.xhtml

were also included in the model. Model 1 includes only age and gender as traditional confounding factors. Model 2 adds smoking status and LTPA as lifestyle factors, which could be regarded as possible confounders or risk factors for outcome. Finally, Model 3 includes hypertension, T2D, and BMI, which could be considered as mediators or strong determinants of diastolic dysfunction.

By presenting all three models the reader will have the possibility to judge the extent to which a change of the effect measure reflects removing a confounding effect and the extent to which the change of the effect measure reflects the degree of possible mediation.

These associations were expressed as odds ratios (OR) with 95% confidence intervals (CI). All tests were 2-sided and statistical significance were assumed at $p < 0.05$.

Ethical considerations

The investigation conforms to the principles outlined in the Declaration of Helsinki. The research ethics committee at the University of Gothenburg approved the study (DNR Ö199-01). The participants provided written informed consent to their participation in the health survey, and a further written informed consent was obtained before undergoing an ultrasound-Doppler examination of the heart.

Patient and Public Involvement

A meeting in the study center and the primary health care center was organized priory to the start of the study. A discussion with caregivers was organized to meet their concerns and ideas for the study but also to organize the care for individuals with pathological findings in the study. An open house event for the public was organized to inform and receive questions and suggestions. The results of the study are disseminated within the health care centers and health professionals.

Data availability statement

The data generated in the current study are available upon reasonable request, considering the sensitivity and confidentiality of the data. Due to the nature of the data, which contains personally identifiable information and sensitive information, it cannot be openly shared in a public repository.

For peer review only

Results

The mean age of the population was 55.4±13 years. In total, 79 of 500 men (16.5%) and 58 of 538 (12.9%) women were diagnosed with diastolic dysfunction (p=0.037). A total of 94% of the cases were categorized as *impaired relaxation* (74 men, 55 women) and 6% as *pseudonormal* (5 men, 3 women). There were no cases of severe *restrictive* filling patterns.

Alcohol consumption was common in both genders; men 84.5% (412/486) and women 68.8% (375/527), however, significant differences were observed between men and women with women being non-drinkers to a larger extent. In this observational study, non-drinkers were older, had higher blood pressure and had more often diabetes, however. Being allocated in one of groups based on alcohol consumption was not associated with diastolic dysfunction (p=0.500)*(Table1)*. Meanwhile, among subjects not using alcohol, a higher rate of left ventricular hypertrophy (LVH) was observed. Characteristics of the study population as well as echocardiographic characteristics related to level of alcohol intake are presented in *Table 1*.

Table 1 Characteristics of participants related to level of alcohol consumption.

Variables	alcohol consumption			
	Non consumers n=226 (22%)	Low n=531 (53%)	Medium-High n=254 (25%)	P*
Diastolic dysfunction[a]	35(15.5%)	66 (12.4%)	32 (12.6%)	0.500
Age (years)[b]	55.4(12.8)	49.5(11.5)	48 (10.7)	0.050
Women[a]	152 (67.3%)	289 (54.4%)	85 (33.5%)	<0.001
BMI (kg/m2)[b]	27.8(5.4)	26.8(4.2)	27 (3.9)	<0.001
SBP (mmHg)[b]	128.4(18.9)	123.6(17.1)	123.2 (15.6)	0.001
DBP (mmHg)[b]	71.1 (9.7)	71.1 (10.3)	72.1 (10.1)	0.300
HDL (mmol/L)[b]	1.2(0.3)	1.3(0.3)	1.3 (0.3)	0.100
TG (g/L)[b]	1.4(0.7)	1.3(0.8)	1.3 (0.7)	0.700
Level of physical activities				0.020
mobile	57(25.2%)	191(36.0%)	90(35.4%)	
medium	147(65%)	312(58.8%)	149(58.7%)	
sedentary	22(9.7%)	28(5.3%)	12(5.9%)	
Hypertension[a]	61 (27.0%)	93 (17.5%)	33 (13%)	<0.001
Diabetes[a]	29 (12.8%)	34 (6.4%)	10 (3.9%)	<0.001
Smoking habits[a]	39 (17.3%)	92 (17.3%)	54 (21.4%)	0.300
E/A ratio	1.2(0.4)	1.4(0.4)	1.4(0.4)	<0.001
IVRT	98.4(21.9)	91.6(20.1)	93.2(21.2)	<0.001
EJECTION FRACTION	72.2(9.6)	74.4(8.4)	74.4(7.8)	0.005
Septal wall (cm)	1(0.2)	0.9(0.2)	0.9(0.2)	0.008

For peer review only - http://bmjopen.bmj.com/site/about/guidelines.xhtml

Posterior wall (cm)	1(0.3)	0.9(0.2)	1(0.5)	0.010
LAD (cm)	3.3(0.5)	3.2(0.5)	3.3(0.5)	0.040
LV-mass (Median and 5th, 95th percentiles)	37.8 (23.4, 62)	34.5 (23.8, 55.6)	35.1 (23.9, 57.5)	0.050*
LVH	52 (23.0%)	70(13.2%)	32(12.6%)	0.001#

[a] data is presented as counts (percent).[b] data are presented with mean (standard deviation) *P values are based ANOVA if the variables are continuous or based on chi-square test if the variables are binary. Medium-high is defined as consumption >56 g/week and low those with alcohol consumption ≤56g/week. BMI, Body mass index, SBP, Systolic blood pressure, DBP Diastolic blood pressure, HDL, High density lipoprotein- cholesterol, TG, Triglycerides, IVRT Isovolumic relaxation time, LAD-left atrial diameter

In a multivariable theoretical model, adjusted for possible confounders and mediators in the association between alcohol consumption and diastolic dysfunction, we found that alcohol consumption was significantly associated with increased risk of diastolic dysfunction. Interestingly, even low alcohol consumption was strongly associated with increased risk for diastolic dysfunction (OR=2.2 95% CI 1.3-3.9). The estimates were even higher for participants with moderate to high alcohol consumption (OR=3.1 95% CI 1.6-6.2) with a strong significant trend (p=0.001). Similar results were observed when adjustments for systolic blood pressure were included in the final models (Low consumption: OR= 2.3 95% CI 1.3-4.1; moderate to high alcohol consumption OR= 3.1 95% CI 1.6-6.2). In another model we tested also to adjust in the final model for HDL-cholesterol on the top of adjustments for age, gender, hypertension, type 2 diabetes, BMI, smoking habits and leisure time physical activities and the association was still strong and significant (Low consumption: OR= 2.4 95% CI 1.4-4.4; moderate to high alcohol consumption OR= 3.4 95% CI 1.7-6.9). Exploring in the same models the association between alcohol consumption and left atrial diameter no significant differences were observed between group with no consumption and groups with low and moderate to high (Age and sex adjustments: low consumption: mean difference(cm) =-0.04 95% CI -0.11 - 0.02; moderate to high alcohol consumption mean difference(cm) =-0.02 95% CI -0.10 - 0.07; fully adjusted model: low consumption: mean difference(cm) =-0.02 95% CI -0.08 - 0.04; moderate to high alcohol consumption mean difference(cm) =0.0 95% CI -0.07 -

For peer review only - http://bmjopen.bmj.com/site/about/guidelines.xhtml

0.08). There were no significant differences in these associations were observed between men and women (p for interaction >0.25). High age, male sex, hypertension, high BMI, and a low level of leisure time physical activity were all also independently associated with diastolic dysfunction (*Table 2)*.

Table 2: Association between risk factors and diastolic dysfunction

	OR	95% confidence interval
**Model 1**		
Age	1.16	1.13-1.19
Men vs Women	1.34	0.87-2.07
Alcohol consumption		
None	1	Ref
Low vs none	1.82	1.08-3.12
High vs none	2.39	1.26-4.58
**Model 2**		
Age	1.16	(1.13, 1.19)
Men vs Women	1.46	(0.94, 2.27)
Current smoking	1.39	(0.78, 2.42)
physical activity		
active	1	Ref
medium vs active	1.6	(0.97, 2.69)
sedentary vs active	4.4	(1.93, 9.96)
alcohol consumption	1	Ref
low vs non	2.03	(1.19, 3.5)
high vs non	2.68	(1.4, 5.19)
**Model 3**		
Age	1.14	(1.11-1.18)
Men vs Women	1.63	(1.02-2.63)
BMI	1.07	(1.01-1.11)
Hypertension	2.68	(1.65-4.33)
Type 2 Diabetes	1.88	(1.00-3.53)
Current smoking	1.76	(0.96-3.15)
Leisure time physical activity		
medium vs active	1.28	(0.75-2.23)
sedentary vs active	2.82	(1.15-6.84)
Alcohol consumption		
None	1	Ref
low vs none	2.25	(1.29-4.02)
high vs none	3.06	(1.55-6.15)

For peer review only

Multivariable logistic regression analyses are used to investigate the association between explanatory variables and diastolic dysfunction. OR Odds ratio, BMI body mass index. In model 1 adjustments for age and gender, in model 2 adjustments for age, gender, current smoking, leisure time physical activities, in model 3 adjustments for age, gender, BMI, hypertension, type 2 diabetes, current smoking, leisure time physical activities are computed. Women are the reference group in the analyses men vs women and physically active group is the reference in the analyses of leisure time physical activity

Similar analyses were computed using LVH as outcome, but no significant association was found with alcohol consumption (*Table3*).

Table 3: Association between risk factors and left ventricular hypertrophy

	OR	confidence interval
Age	1.08	(1.06-1.1)
Men vs Women	1.16	(0.75-1.78)
BMI	1.16	(1.11-1.22)
Hypertension	3.20	(2.05-5.01)
Type 2 Diabetes	1.30	(0.70-2.38)
Current smoking	1.53	(0.89-2.57)
Leisure time physical activity		
medium vs mobile	0.96	(0.60-1.56)
sedentary vs mobile	1.18	(0.50-2.69)
Alcohol consumption		
Non drinkers	1	Ref
low vs non	1.08	(0.59-1.98)
high vs non	1.53	(0.89-2.57)

Multivariable logistic regression analyses are used to investigate the association between explanatory variables and left ventricular hypertrophy. OR Odds ratio, BMI body mass index

For peer review only

For peer review only - http://bmjopen.bmj.com/site/about/guidelines.xhtml

Discussion

In this cross-sectional observational study, we found a strong and independent association between self-reported alcohol consumption and diastolic dysfunction. Interestingly, our data show an increased risk of having diastolic dysfunction already in subjects with low-level of alcohol consumption. Moreover, in gender specific analyses no gender differences were observed suggesting a similar effect of alcohol in both men and women. Despite these findings, no significant association between alcohol intake and LVH were observed. Like previous observations, age, gender, hypertension, leisure time physically activity levels, and BMI were all strongly and independently associated with diastolic dysfunction.

These findings are not in line with most of the observations on the role of alcohol in heart failure. In fact, population-based studies have shown that *moderate* levels of alcohol consumption (i.e. <1 drinks per day (d/d) in females and <2 d/d in males) have a protective effect on ischemic heart disease and decreases the incidence of CHF (1, 28) (3). The beneficial effect of a low to moderate amount of alcoholic intake are not reported by all epidemiological studies (29, 30). A 'healthy cohort effect' is reported as one plausible explanation to the favourable effects of moderate consumption (29, 30) where the confounding effects of for example physical activity, mental health, psychosocial, socioeconomic and social network related factors seldom are accounted for (30). Moreover, in a recent observation in HUNT study (31) no protective effect of low-alcohol consumption on left ventricular function was observed, however, a positive linear association was observed between alcohol consumption and left ventricular mass and end diastolic diameter. The findings of HUNT-study are in line with our findings however the significance in the association was lost in their multivariable analyses. Interestingly, before the adjustments in our cohort higher prevalence of diastolic dysfunction was observed in nondrinkers. These differences were not significant. The same group had also higher age, lower level of physical activities, higher systolic blood pressure and higher prevalence of hypertension and diabetes. It was first after the adjustments for these factors that a positive linear association between alcohol consumption and diastolic dysfunction was observed. These findings suggest no protective effect of alcohol at low

For peer review only - http://bmjopen.bmj.com/site/about/guidelines.xhtml
For peer review only

levels. Also, alcohol seems not to be related with better diastolic dysfunction, but healthy and younger subjects are more prone to drink alcohol and they have of course better diastolic function but not of alcohol consumption.

Several alcohol metabolites are identified as particularly toxic to the myocardium: ethanol, acetaldehyde (the metabolic product of alcohol) and fatty acid ethyl esters (32, 33). Alcohol also impairs the transport of calcium ions from the sarcoplasma to the sarcoplasmatic reticulum (34, 35) resulting in diastolic dysfunction characterized by delayed myocardial relaxation (36) and impaired left ventricular (LV) diastolic filling. Moderate versus high doses of alcohol affect myocardial function differently through oxidative stress, haemostatic factors, levels of C-reactive protein, interleukins, and TNF alpha (29).

There have been observations that suggest that patterns of drinking and food intake might explain at least partially the damages of alcohol in myocardium(5, 37, 38). In fact, the Mediterranean countries with a low prevalence of CVD, alcohol is commonly consumed with meals as compared to Russia with a high prevalence of CVD where binge drinking is much more frequent (30). However, some studies have shown there is evidence that alcoholic cardiomyopathy can be developed even in absence of malnutrition(39) while these deleterious effects are exaggerated of malnutrition(40, 41).

Interestingly, our results did not show any significant association with LVH. One reason of the lack of association might be the relatively low number of individuals with high consumption in this cohort. Measurable structural remodelling might need higher level of alcohol consumption. In fact, a large population study in South Korea with significantly larger number of participants compared with our study, could find a strong association between alcohol consumption and LVH(42).

Strengths and limitations

Echocardiography was used in this large population-based cohort using standardized age-dependent criteria to identify diastolic disfunction based on recommended by international expert groups. The re-evaluation of a sub-sample by a senior cardiologist confirmed the first assessment and the consistency in assessment of prevalence of diastolic dysfunction and in identified risk factors between our findings and other

For peer review only - http://bmjopen.bmj.com/site/about/guidelines.xhtml

For peer review only

comprehensive surveys using Doppler techniques, lend further support to the validity of our data.

The characteristics of alcohol consumers and prevalence of alcohol consumption was in accordance with other comparable community-based surveys (43, 44). This, however, might be a source of underestimation of the alcohol consumption. In fact, in several studies there is an underestimation of self-reported alcohol consumption(45) and there is need of more objective and reliable measures of alcohol consumption. However, the underestimation should be more frequent and mostly in the group with higher consumption. This would not have an impact effect on our results in the association found between low alcohol consumption and diastolic dysfunction. Moreover, the findings are based on a rural population in Sweden and differences in these association might appear when other population are considered however the consistency of the results with previous findings indicate that these findings might apply in other populations. Finally, this was a cross sectional observation and no conclusions in causality can be drawn and the residual confounding is not possible to rule out.

Conclusion

Our study revealed a significant association between alcohol consumption and diastolic dysfunction starting already at low levels that was supported by a dose-dependent pattern. Additional prospective surveys with adequate measurement of alcohol consumption and possible confounders are needed to improve our understanding of the underlying relationships and mechanisms. Our results might be of importance in the development of public health policies and preventive strategies.

Competing interests

The authors declare that they have no competing interests.

Acknowledgements and funding

We are indebted to all participants in the Vara Cohort. Without them this study would not been possible. The Knut and Alice Wallenberg foundation is recognized for generous support. This work was supported by: The Swedish Science Council; Sahlgrenska Academy, University of Gothenburg; Skaraborg Institute; The Health & Medical Care

For peer review only

Committee of the Regional Executive Board of the Region Västragötalands region VGFOUREG-86294, VGFOUREG-969459, ALFGBG-966255, ALFGBG-971147; Skaraborg Primary Care; Skane Region; Lund University; University Hospital of Malmoe donation Foundation; Påhlsson Foundation; Crafoord Foundation; Lundberg Foundation, the Swedish Heart and Lung foundation and the Wallenberg Center for Molecular Medicine, Lund University; and Lund University. The funding source was not involved in the work with the article.

Author contribution: All authors read and approved the final manuscript. Research idea and study design: LB, BD, UL, MH; Data acquisition: UL, LR, BD, MH; Data analysis and interpretation: LB, UL, BD, MM, MH; Statistical analyses: LB, BD, YL, UL. Each author contributed important intellectual content during manuscript drafting or revision, accepts personal accountability for the author's own contributions, and agrees to ensure that questions pertaining to the accuracy or integrity of any portion of the work are appropriately investigated and resolved.

For peer review only

References

1.Djousse, L. and Gaziano, J.M. (2007). Alcohol consumption and risk of heart failure in the Physicians' Health Study I. Circulation 115: 34-39.

2.World Health Organization. Management of Substance Abuse Team. Global status report on alcohol and health, pvolumes, World Health Organization, Geneva, Switzerland.

3.Zhong, L., Chen, W., Wang, T., Zeng, Q., Lai, L., Lai, J., Lin, J. and Tang, S. (2022). Alcohol and Health Outcomes: An Umbrella Review of Meta-Analyses Base on Prospective Cohort Studies. Front Public Health 10: 859947.

4.Larsson, S.C., Wallin, A. and Wolk, A. (2018). Alcohol consumption and risk of heart failure: Meta-analysis of 13 prospective studies. Clin Nutr 37: 1247-1251.

5.Murray, R.P., Connett, J.E., Tyas, S.L., Bond, R., Ekuma, O., Silversides, C.K. and Barnes, G.E. (2002). Alcohol volume, drinking pattern, and cardiovascular disease morbidity and mortality: is there a U-shaped function? Am J Epidemiol 155: 242-248.

6.Lazarevic, A.M., Nakatani, S., Neskovic, A.N., Marinkovic, J., Yasumura, Y., Stojicic, D., Miyatake, K., Bojic, M. and Popovic, A.D. (2000). Early changes in left ventricular function in chronic asymptomatic alcoholics: relation to the duration of heavy drinking. J Am Coll Cardiol 35: 1599-1606.

7.(1998). How to diagnose diastolic heart failure. European Study Group on Diastolic Heart Failure. Eur Heart J 19: 990-1003.

8.Fischer, M., Baessler, A., Hense, H.W., Hengstenberg, C., Muscholl, M., Holmer, S., Doring, A., Broeckel, U., Riegger, G. and Schunkert, H. (2003). Prevalence of left ventricular diastolic dysfunction in the community. Results from a Doppler echocardiographic-based survey of a population sample. Eur Heart J 24: 320-328.

9.Redfield, M.M., Jacobsen, S.J., Burnett, J.C., Jr., Mahoney, D.W., Bailey, K.R. and Rodeheffer, R.J. (2003). Burden of systolic and diastolic ventricular dysfunction in the community: appreciating the scope of the heart failure epidemic. JAMA 289: 194-202.

10.Vogel, M.W., Slusser, J.P., Hodge, D.O. and Chen, H.H. (2012). The natural history of preclinical diastolic dysfunction: a population-based study. Circ Heart Fail 5: 144-151.

11.Catena, C., Colussi, G., Verheyen, N.D., Novello, M., Fagotto, V., Soardo, G. and Sechi, L.A. (2016). Moderate Alcohol Consumption Is Associated With Left Ventricular Diastolic Dysfunction in Nonalcoholic Hypertensive Patients. Hypertension 68: 1208-1216.

12.Liu, F., Liu, Y., Sun, X., Yin, Z., Li, H., Deng, K., Zhao, Y., Wang, B., Ren, Y., Liu, X., Zhang, D., Chen, X., Cheng, C., Liu, L., Liu, D., Chen, G., Hong, S., Wang, C., Zhang, M. and Hu, D. (2020). Race- and sex-specific association between alcohol consumption and hypertension in 22 cohort studies: A systematic review and meta-analysis. Nutr Metab Cardiovasc Dis 30: 1249-1259.

13.Chakraborty, S. (2014). Analysis of NHANES 1999-2002 data reveals noteworthy association of alcohol consumption with obesity. Ann Gastroenterol 27: 250-257.

14.Larsson, C.A., Kroll, L., Bennet, L., Gullberg, B., Rastam, L. and Lindblad, U. (2012). Leisure time and occupational physical activity in relation to obesity and insulin resistance: a population-based study from the Skaraborg Project in Sweden. Metabolism 61: 590-598.

For peer review only - http://bmjopen.bmj.com/site/about/guidelines.xhtml

15.Ingelsson, E., Bennet, L., Ridderstrale, M., Soderstrom, M., Rastam, L. and Lindblad, U. (2008). The PPARGC1A Gly482Ser polymorphism is associated with left ventricular diastolic dysfunction in men. BMC Cardiovasc Disord 8: 37.
16.(1999). 1999 World Health Organization-International Society of Hypertension Guidelines for the Management of Hypertension. Guidelines Subcommittee. J Hypertens 17: 151-183.
17.Alberti, K.G. and Zimmet, P.Z. (1998). Definition, diagnosis and classification of diabetes mellitus and its complications. Part 1: diagnosis and classification of diabetes mellitus provisional report of a WHO consultation. Diabet Med 15: 539-553.
18.Goransson, M. and Hanson, B.S. (1994). How much can data on days with heavy drinking decrease the underestimation of true alcohol consumption? J Stud Alcohol 55: 695-700.
19.Bantle, J.P., Wylie-Rosett, J., Albright, A.L., Apovian, C.M., Clark, N.G., Franz, M.J., Hoogwerf, B.J., Lichtenstein, A.H., Mayer-Davis, E., Mooradian, A.D. and Wheeler, M.L. (2006). Nutrition recommendations and interventions for diabetes--2006: a position statement of the American Diabetes Association. Diabetes Care 29: 2140-2157.
20.Nyholm, M., Gullberg, B., Haglund, B., Rastam, L. and Lindblad, U. (2008). Higher education and more physical activity limit the development of obesity in a Swedish rural population. The Skaraborg Project. Int J Obes (Lond) 32: 533-540.
21.Lang, R.M., Bierig, M., Devereux, R.B., Flachskampf, F.A., Foster, E., Pellikka, P.A., Picard, M.H., Roman, M.J., Seward, J., Shanewise, J.S., Solomon, S.D., Spencer, K.T., Sutton, M.S. and Stewart, W.J. (2005). Recommendations for chamber quantification: a report from the American Society of Echocardiography's Guidelines and Standards Committee and the Chamber Quantification Writing Group, developed in conjunction with the European Association of Echocardiography, a branch of the European Society of Cardiology. J Am Soc Echocardiogr 18: 1440-1463.
22.Jarnert, C., Mejhert, M., Ring, M., Persson, H. and Edner, M. (2000). Doppler tissue imaging in congestive heart failure patients due to diastolic or systolic dysfunction: a comparison with Doppler echocardiography and the atrio-ventricular plane displacement technique. Eur J Heart Fail 2: 151-160.
23.Gudmundsson, P., Rydberg, E., Winter, R. and Willenheimer, R. (2005). Visually estimated left ventricular ejection fraction by echocardiography is closely correlated with formal quantitative methods. Int J Cardiol 101: 209-212.
24.Oh, J.K., Appleton, C.P., Hatle, L.K., Nishimura, R.A., Seward, J.B. and Tajik, A.J. (1997). The noninvasive assessment of left ventricular diastolic function with two-dimensional and Doppler echocardiography. J Am Soc Echocardiogr 10: 246-270.
25.Swedberg, K., Cleland, J., Dargie, H., Drexler, H., Follath, F., Komajda, M., Tavazzi, L., Smiseth, O.A., Gavazzi, A., Haverich, A., Hoes, A., Jaarsma, T., Korewicki, J., Levy, S., Linde, C., Lopez-Sendon, J.L., Nieminen, M.S., Pierard, L. and Remme, W.J. (2005). Guidelines for the diagnosis and treatment of chronic heart failure: executive summary (update 2005): The Task Force for the Diagnosis and Treatment of Chronic Heart Failure of the European Society of Cardiology. Eur Heart J 26: 1115-1140.
26.de Simone, G., Devereux, R.B., Chinali, M., Roman, M.J., Lee, E.T., Resnick, H.E. and Howard, B.V. (2009). Metabolic syndrome and left ventricular hypertrophy in the prediction of cardiovascular events: the Strong Heart Study. Nutr Metab Cardiovasc Dis 19: 98-104.

For peer review only - http://bmjopen.bmj.com/site/about/guidelines.xhtml

27.Krauser, D.G. and Devereux, R.B. (2006). Ventricular hypertrophy and hypertension: prognostic elements and implications for management. Herz 31: 305-316.
28.Walsh, C.R., Larson, M.G., Evans, J.C., Djousse, L., Ellison, R.C., Vasan, R.S. and Levy, D. (2002). Alcohol consumption and risk for congestive heart failure in the Framingham Heart Study. Ann Intern Med 136: 181-191.
29.Bau, P.F., Bau, C.H., Rosito, G.A., Manfroi, W.C. and Fuchs, F.D. (2007). Alcohol consumption, cardiovascular health, and endothelial function markers. Alcohol 41: 479-488.
30.Fuchs, F.D. and Chambless, L.E. (2007). Is the cardioprotective effect of alcohol real? Alcohol 41: 399-402.
31.Gemes, K., Janszky, I., Strand, L.B., Laszlo, K.D., Ahnve, S., Vatten, L.J., Dalen, H. and Mukamal, K.J. (2018). Light-moderate alcohol consumption and left ventricular function among healthy, middle-aged adults: the HUNT study. BMJ Open 8: e020777.
32.Beckemeier, M.E. and Bora, P.S. (1998). Fatty acid ethyl esters: potentially toxic products of myocardial ethanol metabolism. J Mol Cell Cardiol 30: 2487-2494.
33.Ren, J. and Wold, L.E. (2008). Mechanisms of alcoholic heart disease. Ther Adv Cardiovasc Dis 2: 497-506.
34.Cofan, M., Nicolas, J.M., Fernandez-Sola, J., Robert, J., Tobias, E., Sacanella, E., Estruch, R. and Urbano-Marquez, A. (2000). Acute ethanol treatment decreases intracellular calcium-ion transients in mouse single skeletal muscle fibres in vitro. Alcohol Alcohol 35: 134-138.
35.Noren, G.R., Staley, N.A., Einzig, S., Mikell, F.L. and Asinger, R.W. (1983). Alcohol-induced congestive cardiomyopathy: an animal model. Cardiovasc Res 17: 81-87.
36.Kupari, M., Koskinen, P., Suokas, A. and Ventila, M. (1990). Left ventricular filling impairment in asymptomatic chronic alcoholics. Am J Cardiol 66: 1473-1477.
37.Urquiaga, I., Guasch, V., Marshall, G., San Martin, A., Castillo, O., Rozowski, J. and Leighton, F. (2004). Effect of Mediterranean and Occidental diets, and red wine, on plasma fatty acids in humans. An intervention study. Biol Res 37: 253-261.
38.Britton, A. and McKee, M. (2000). The relation between alcohol and cardiovascular disease in Eastern Europe: explaining the paradox. J Epidemiol Community Health 54: 328-332.
39.Urbano-Marquez, A., Estruch, R., Navarro-Lopez, F., Grau, J.M., Mont, L. and Rubin, E. (1989). The effects of alcoholism on skeletal and cardiac muscle. N Engl J Med 320: 409-415.
40.Fernandez-Sola, J. (2015). Cardiovascular risks and benefits of moderate and heavy alcohol consumption. Nat Rev Cardiol 12: 576-587.
41.Piano, M.R. and Phillips, S.A. (2014). Alcoholic cardiomyopathy: pathophysiologic insights. Cardiovasc Toxicol 14: 291-308.
42.Park, S.K., Moon, K., Ryoo, J.H., Oh, C.M., Choi, J.M., Kang, J.G., Chung, J.Y. and Young Jung, J. (2018). The association between alcohol consumption and left ventricular diastolic function and geometry change in general Korean population. Eur Heart J Cardiovasc Imaging 19: 271-278.
43.Bostrom, G. and Nykvist, K. (2004). Lifestyle and health (in Swedish), The National Institute of Public Health in Sweden. Available at *http://www.fhi.se/upload/ar2005/rapporter/r200448levnadsvanorhalsa0504.pdf*

For peer review only - http://bmjopen.bmj.com/site/about/guidelines.xhtml

44. Wilsgaard, T. and Jacobsen, B.K. (2007). Lifestyle factors and incident metabolic syndrome. The Tromso Study 1979-2001. Diabetes Res Clin Pract 78: 217-224.
45. Stockwell, T., Zhao, J., Sherk, A., Rehm, J., Shield, K. and Naimi, T. (2018). Underestimation of alcohol consumption in cohort studies and implications for alcohol's contribution to the global burden of disease. Addiction 113: 2245-2249.

For peer review only

Figure 1

Figure 1. Study population in the Vara Cohort 2002-2003 Vara Cohort 1811 participants

*Cases excluded due to at least one of the following reasons: arrhythmia/atrial fibrillation n=8, physical characteristics hindering UCG n=6, self-reported history of heart failure n=6, EF<45% n=2 and/or aortic insufficiency n=2.

For peer review only

Figure 1. Study population in the Vara Cohort 2002-2003

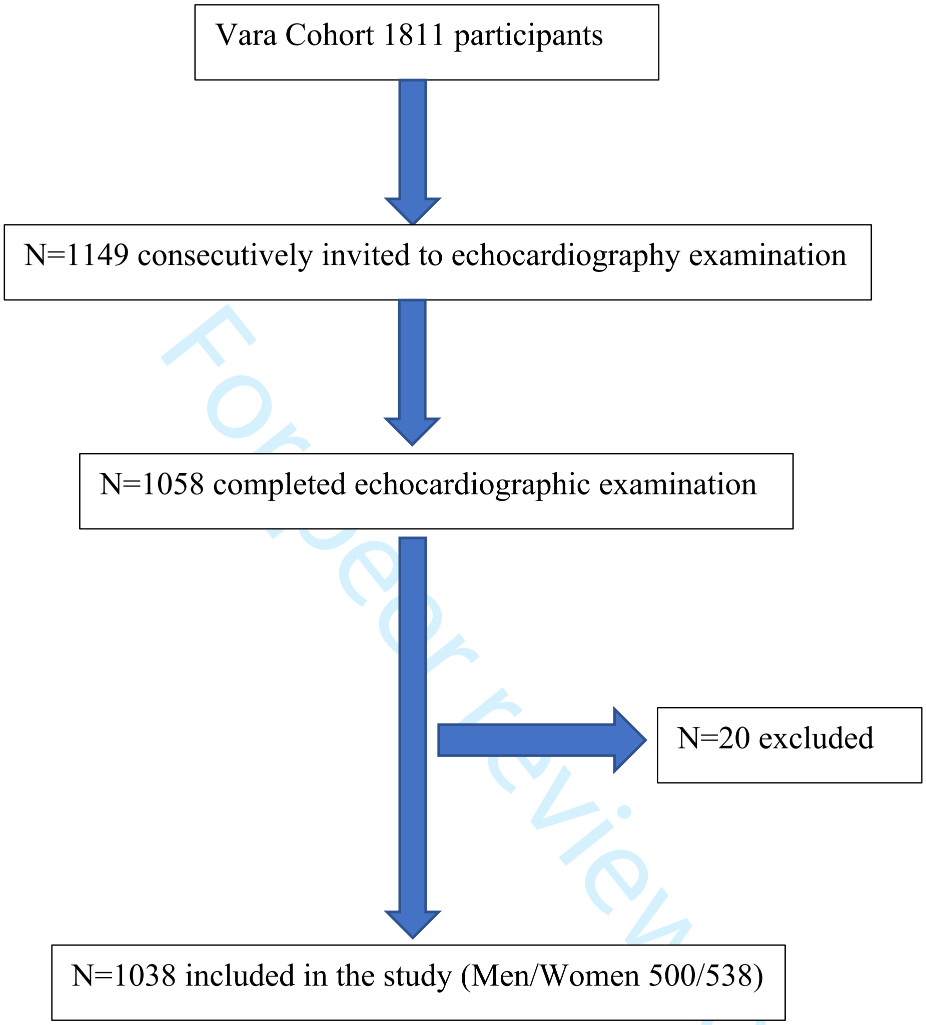

*Cases excluded due to at least one of the following reasons: arrhythmia/atrial fibrillation n=8, physical characteristics hindering UCG n=6, self-reported history of heart failure n=6, EF<45% n=2 and/or aortic insufficiency n=2.

For peer review only
